# Identification of an Additional Metal-Binding Site in Human Dipeptidyl Peptidase III

**DOI:** 10.3390/ijms241612747

**Published:** 2023-08-13

**Authors:** Antonia Matić, Filip Šupljika, Hrvoje Brkić, Jasna Jurasović, Zrinka Karačić, Sanja Tomić

**Affiliations:** 1 Division of Organic Chemistry and Biochemistry, Ruđer Bošković Institute, Bijenička Cesta 54, 10000 Zagreb, Croatia; antonia.matic@irb.hr; 2 Department of Chemistry and Biochemistry, Faculty of Food Technology and Biotechnology, Pierottijeva 6, 10000 Zagreb, Croatia; fsupljika@pbf.hr; 3 Faculty of Medicine, J. J. Strossmayer University of Osijek, 31000 Osijek, Croatia; hbrkic@mefos.hr; 4 Faculty of Dental Medicine and Health, J. J. Strossmayer University of Osijek, 31000 Osijek, Croatia; 5 Analytical Toxicology and Mineral Metabolism Unit, Institute for Medical Research and Occupational Health, 10000 Zagreb, Croatia; jurasovic@imi.hr

**Keywords:** dipeptidyl peptidase III, metal ions, binding affinity, metal inhibition, metal exchange

## Abstract

Dipeptidyl peptidase III (DPP III, EC 3.4.14.4) is a monozinc metalloexopeptidase that hydrolyzes dipeptides from the N-terminus of peptides consisting of three or more amino acids. Recently, DPP III has attracted great interest from scientists, and numerous studies have been conducted showing that it is involved in the regulation of various physiological processes. Since it is the only metalloenzyme among the dipeptidyl peptidases, we considered it important to study the process of binding and exchange of physiologically relevant metal dications in DPP III. Using fluorimetry, we measured the *K*_d_ values for the binding of Zn^2+^, Cu^2+^, and Co^2+^ to the catalytic site, and using isothermal titration calorimetry (ITC), we measured the *K*_d_ values for the binding of these metals to an additional binding site. The structure of the catalytic metal’s binding site is known from previous studies, and in this work, the affinities for this site were calculated for Zn^2+^, Cu^2+^, Co^2+^, and Mn^2+^ using the QM approach. The structures of the additional binding sites for the Zn^2+^ and Cu^2+^ were also identified, and MD simulations showed that two Cu^2+^ ions bound to the catalytic and inhibitory sites exchanged less frequently than the Zn^2+^ ions bound to these sites.

## 1. Introduction

Dipeptidyl peptidase III (DPP III, EC 3.4.14.4) is a monozinc metalloexopeptidase that hydrolyzes dipeptides from the N-terminus of its substrates, consisting of three or more amino acids [1]. Because of its affinity for some bioactive peptides, such as angiotensins and opioid peptides, it has recently attracted the attention of several research groups. As early as 2016, angiotensin-(1-7) was shown to be hydrolyzed by DPP III in renal epithelial cells [2], while Pang et al. revealed a link between DPP III and the renin–angiotensin system (RAS) and, thus the potential use of DPP III in the treatment of hypertension [3]. Recently, Komeno et al. [4] demonstrated the cardio- and reno-protective effects of dipeptidyl peptidase III in diabetic mice. They found that the beneficial role of DPP III is mediated, at least in part, by the cleavage of a cytotoxic peptide, Peptide 2, which was increased in diabetic mice compared with normal mice. In addition, DPP III has recently been proposed as a biomarker for cardiac shock [5]. In its interaction with Keap1, unrelated to its peptidase activity, DPP III is involved in human cancer development, the oxidative-stress response, and neuron protection [6,7,8].

Because of the demonstrated importance of DPP III in the regulation of various physiological processes and because it is the only metalloenzyme among the dipeptidyl peptidases, we considered it important to better understand the process of binding and exchange of metal dications, which are abundant in the human body, in DPP III, and their influence on its structure and function. Abramić et al. [9,10] showed that elevated concentrations of zinc ions (10–30 μM) inhibited rat and human DPP III activity, and Hirose et al. [11] demonstrated the restoration of rat DPP III activity through the addition of either Zn^2+^, Cu^2+^, Ni^2+^, or Co^2+^ to the apoenzyme.

There is only one zinc ion in the active site of the crystallographically determined structures of DPP III (PDB IDs of structures of the following: human—3FVY, 3T6B, 3T6J, 5EGY, 5E2Q, 5E33, 5E3A, 5E3C, 5EHH [12,13]; yeast—3CSK [14], fungal—5YFB, 5YFC, 5YFD [15]; bacterial DPP III—5NA6, 6NA7, 5NA8, 5ZUM, 6EOM [16,17,18]), but previous studies [19,20,21] clearly indicated the possibility of another metal-ion binding, which inhibits the enzymatic activity of DPP III. The binding of another metal ion in the so-called inhibitory metal-binding site, which is directly adjacent to the catalytically active site of the enzyme, has been observed in the crystallographic structures of three zinc-dependent enzymes, in which, as in DPP III, the catalytic zinc is coordinated with two histidines and the carboxyl groups of the amino acids Glu or Asp: carboxypeptidase A, thermolysin, and LpxC (the PDB codes of the corresponding structures are 1CPX, 1LND, and 1P42) [22,23,24]. Young and Siemann showed that in anthrax lethal factor (LF), metal ions are exchanged in such a way that the binding of the metal to the inhibitory binding site precedes the release of catalytic zinc [20]. Using the same procedure that Young and Siemann used to determine the potential binding site of the inhibitory Zn^2+^ ion in LF, we can determine the binding site of the inhibitory metal in DPP III. We have shown computationally that human DPP III (hDPP III) can take up a second zinc ion that binds immediately next to the catalytically important ion and displaces the zinc in the active site, while the zinc that originally occupied the active site leaves the enzyme [19].

By combining several experimental methods (HR-ICP-MS, ITC, stopped-flow, and fluorescence measurements), we studied the stoichiometry and the thermodynamic and kinetic parameters of the binding of various divalent metal ions, Zn^2+^, Cu^2+^, Mn^2+^, and Co^2+^ to purified recombinant human DPP III. In addition, we investigated the binding of the metal ions computationally. Using QM calculations, we determined the thermodynamic parameters for the binding of Zn^2+^, Cu^2+^, Mn^2+^, and Co^2+^ to hDPP III, and using MD simulations, we determined the main binding modes of two Cu ions to hDPP III.

The aim of the proposed research is to increase knowledge about the modes of binding and relative affinity of several physiologically relevant transition metals to dipeptidyl peptidase III and their influence on enzyme activity.

## 2. Results

Several experimental methods were used to determine the stoichiometry and affinity of metal ions binding to DPP III.

### 2.1. Metals in Excess Inactivate DPP III in Stopped-Flow Experiments

The flow curves of the hydrolysis of Arg-Arg-2NA in the presence of different metal ions were determined using stopped-flow instruments by incubating the metal solution with apo hDPP III. Since we failed to obtain an activation or inhibition curve with the apoprotein, we used the native protein (not treated with chelators to remove metal ions).

**Figure 1:**
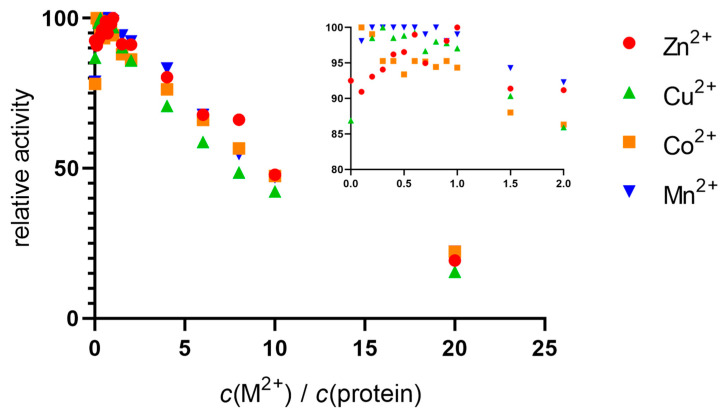
Relative enzyme activity as a function of metal concentration. Using a stopped-flow setup with two syringes, the native protein (10 nM) was incubated with metal solutions (Zn^2+^, Cu^2+^, Co^2+^, and Mn^2+^) in Tris-HCl buffer (40 mM, pH 7.5) and rapidly mixed with the substrate (final concentration of 200 µM), and the progress of the reaction was monitored at 332 nm. Inset: Close-up view of data points at equimolar metal and protein concentrations.

The native hDPP III sample was incubated with a solution of zinc, copper, cobalt, and manganese nitrates. Activation was observed up to a metal:protein ratio of 1:1, after which gradual inactivation occurred. Similar activity curves were obtained for all the metals, with some slight differences in the areas where the ratio of metal to protein was less than 1:1 (see inset in Figure 1). The only metal that showed a peak in activity at equimolar concentrations was Zn^2+^. For the other metals, Cu^2+^, Co^2+^, and Mn^2+^, the activity was generally stable up to a ratio of 1:1, after which inhibition occurred. From the results of the increase in activity, it appeared that the native protein was not saturated with zinc, so we performed measurements using inductively coupled plasma mass spectrometry (ICP-MS).

### 2.2. Dissociation Constants of the Catalytic Binding Site

**Figure 2:**
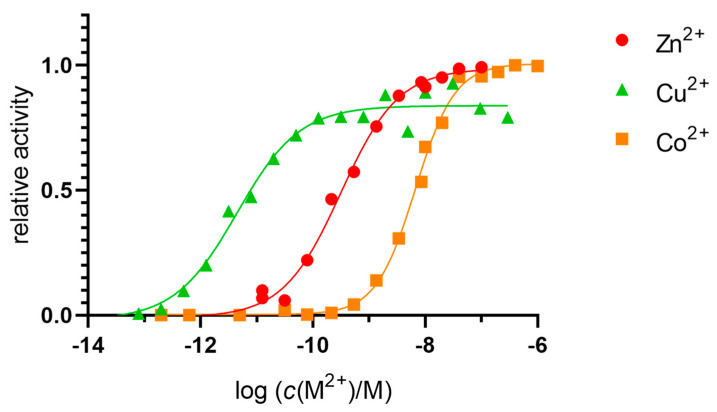
Determination of the dissociation constant of native DPP III. DPP III (10 nM) in 20 mM Tris-HCl buffer pH 7.4 was exposed to metals (Zn^2+^, Cu^2+^, and Co^2+^, 10 µM). An excess of DPA was used to achieve the free metal-ion concentrations indicated in the figure.

The *K*_d_ value for the enzyme was determined by measuring its activity in 20 mM Tris-HCl buffer pH 7.4 with a 1000-fold molar excess of Zn^2+^, Cu^2+^, and Co^2+^ ions (10 µM) compared with the concentration of hDPP III (10 nM) and an excess of DPA as a chelator (Figure 2). The *K*_d_ value (which was identical to the concentrations of the free ions Zn^2+^, Cu^2+^, and Co^2+^ required to reach half of the maximum activity of the enzyme) was determined by non-linear regression and corrected for metal-ion-buffer binding, with final values of 6.7 × 10^−11^ M for the Zn^2+^, 2.8 × 10^−12^ M for the Cu^2+^, and 3.2 × 10^−9^ M for the Co^2+^.

Strong inactivation with excess zinc was also observed. Since no data were available for the binding constants of the manganese and DPA complexes, we could not measure the dissociation constant for the manganese.

### 2.3. Additional Metal-Binding Site Confirmed Using ICP-MS

Measurements were performed in 25 mM ammonium acetate, pH 7.4, at a mass concentration of protein of 0.1 to 0.2 mg mL^−1^. The holoproteins were prepared with the addition of six moles of metal ions to one mole of protein, and with subsequent washing with the buffer to remove the unbound metal ions (see Section 4). The results are shown in Table 1. The native protein was not saturated with zinc metal, as we previously observed in the stopped-flow experiments. Our results show that the native protein was, in fact, very similar to the apoprotein. The highest metal-to-protein ratio measured was 2:1, for copper. Only one mole of Zn^2+^ was bound to one mole of protein. The results confirm that the hDPP III protein has a high affinity for Cu^2+^ and Zn^2+^, leading to integer values for the metal-to-protein content, while Co^2+^ and Mn^2+^ do not remain tightly bound; values below 1 were observed, specifically about 0.3 for cobalt and 0.02–0.03 for manganese. The results were reproducible with different batches of protein. These results confirm the possibility of an additional metal-ion-binding site in hDPP III. Subsequently, ITC measurements were performed to describe the thermodynamics of the binding of these metals.

### 2.4. Dissociation Constants of the Additional Metal-Binding Side

**Figure 3:**
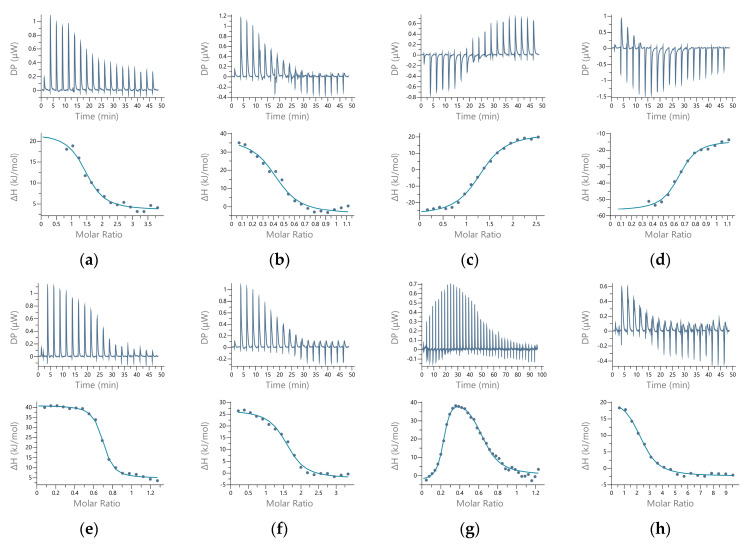
Titration of human DPP III with metal ions in 50 mM sodium cacodylate buffer, pH 7.4. Binding isotherms (upper panel) and data with fitting curves (lower panel) are shown (left to right) for direct (metal to protein) and reverse (protein to metal) titrations, respectively, for Zn^2+^ (**a**,**b**), Cu^2+^ (**c**,**d**), Co^2+^ (**e**,**f**), and Mn^2+^ (**g**,**h**).

We used isothermal titration calorimetry (ITC) to quantify the metal–protein interactions. This method should provide a description of all the thermodynamic parameters of the interaction, but careful experimentation and data processing are required. All the titrations of hDPP III with the metal ions were tested as reverse titrations (protein in metal ion). The main results are shown in Figure 3.

The overall results indicated two binding sites (Table 2). For all the metals except cobalt, the data indicated at least two binding sites, based either on the stoichiometry (*n*) or on the shape of the curve. The affinity of the active site for Zn^2+^ and Cu^2+^ was, theoretically, too large to be measured by this method. However, for Co^2+^, we measured the binding of 0.63 metal ions per protein molecule. This binding was endothermic (Figure 3c), and the *K*_d_ (corrected for the interaction of the metal ion with the buffer, and the buffer protonation) was 13 nM (Table 3), as expected for the binding of the Co^2+^ to the active protein site. The data from the reverse titration were in agreement with the direct titration (Tables S1 and S2). For manganese, the data were fitted to two sets of sites, but with a total metal content per protein of less than 1. For the first (active) binding site, the apparent *K*_d_ value was similar to that for cobalt, but for the second (additional) binding site, it was significantly higher. The binding to the first site was exothermic, while to the second site, it was endothermic. For zinc, we could not obtain the first baseline, with the data suggesting two binding sites, similar to manganese. In these cases, we eliminated all the points that were potentially associated with another binding event and analyzed the data using “one set of sites” fitting. The stoichiometry was 1.5 zinc ions per protein molecule for direct titration and 2.5 for reverse titration. Results could only be obtained for the binding to the second site, as the first site bound the Zn^2+^ with much larger affinity. For all of these metals, the data from the direct and reverse titrations were in general agreement.

For copper, we did not obtain satisfactory results. The direct and reverse titrations of protein and Cu^2+^ did not match exactly, which can be observed immediately on the signature plot (Figure S1). Both binding processes were exothermic, but the second had a significant entropic effect. Although both titrations indicated that we measured binding to the same—additional—binding site (based on the overall stoichiometry *n* > 1), the reverse titration showed an additional binding event. We suspect that there may have been other interactions in the reaction (e.g., Cu^2+^–buffer, or Cu^2+^–His-tag) that interfered with the binding of the metal to the hDPP III, so we cannot consider the data obtained to have been accurate. The amount of His-tag remaining in the protein sample was determined using immunoassays; the data are given in the supplementary material (Figures S2 and S3).

The measurements were repeated in the 50 mM MOPS-NaOH buffer at pH 7.4 for Zn^2+^ and Cu^2+^ (see Table S2). Again, we could not use the data obtained with the copper ions because the direct and reverse titrations did not match. However, the apparent parameters measured in the direct titration in both buffers agreed, with the largest difference observed in the stoichiometry (*n* = 1.2 in the sodium cacodylate buffer and *n* = 0.8 in MOPS-NaOH).

Mutants were made to test the predictions about the amino acid residues forming the additional binding site. We tested the E508D variant and the E316A H568Y double mutant. Overall, we found no significant differences in the binding of the zinc ions to the wild type and variants of hDPP III, either in the stoichiometry nor in the apparent *K*_d_ values (Table S3). However, the apparent *K*_d_ value increased significantly for the copper ions (Table S4). From these data, we can surmise that we indeed measured the binding of metal ions to a protein-binding site in the direct titration experiments. Therefore, we interpreted the data from the direct titrations as showing the binding of Cu^2+^ to the additional metal-binding site (data in Table 2). In the reverse titration with the Cu^2+^, we probably measured an unknown, competing interaction.

The apparent *K*_d_ values (Table 2) were modified using the known constants for metal–buffer interactions. The *K*_d_ values thus determined are given in Table 3. The cobalt ions bound only to the active site, and the *K*_d_ value from the ITC data indicated an affinity close to nM, which was consistent with the data from the fluorimetric assay. The *K*_d_ values for the binding of the zinc and copper to the additional binding site were in the order of 10^−7^ M and 10^−8^ M, respectively. These affinities were four orders of magnitude lower than the fluorimetrically measured affinities for the catalytic site.

### 2.5. Computational Approach

#### 2.5.1. Affinity of Different Metal Cations for the Catalytically Active Metal-Binding Site: Quantum Mechanical (QM) Calculations

We performed QM calculations on simplified models of the hDPP III metal center to determine whether other divalent cations, such as Cu^2+^, Co^2+^, and Mn^2+^, can replace native zinc and how the presence of water molecules and the amino acid residues of the second coordination sphere affect this process.

Since higher concentrations of these metal ions have been shown to inhibit the enzymatic activity of hDPP III, we also studied the binding of the Zn^2+^ and the Cu^2+^ in both the catalytic and an additional binding site next to the catalytic site, when the same type of metal was bound in both sites.

Selected distances in the hDPP III structures: model 1, model 2, model 3, and model 4 with metal ions (Zn, Cu, Co, and Mn) before (I) and after the energy optimization are given in Tables S5–S8.

To determine the competition between the cognate Zn^2+^ and the other biogenic metal species, such as Cu^2+^, Co^2+^, and Mn^2+^, for the active metal-binding site in the hDPP III, the relative Gibbs free energies were calculated according to Equation (1):

ΔG = ΔG ([M1 + P]) − ΔG ([M2 + P]) − ΔG ([M1 + water]) + ΔG([M2 + water]) (1)

where M1 represents the cognate Zn^2+^, M2 is either the Cu^2+^, Co^2+^, or Mn^2+^ and P is the protein (see Table 4).

The enzyme active site itself was represented by three models with different levels of complexity (see Section 4).

The relative Gibbs free energy for the binding of the Cu to the inhibitory active site when the active site was occupied was calculated as 28.68 kcal mol^−1^, according to the following equation:

ΔG = ΔG ([2M1 + P]) − ΔG ([2M2 + P]) − ΔG ([M1 + P]) − ΔG ([M1 + water]) + ΔG ([M2 + P]) + ΔG ([M2 + water])(2)

where M1 represents the cognate Zn^2+^, M2 is either the Cu^2+^, the Co^2+^, the or Mn^2+^, and P is the protein.

#### 2.5.2. Molecular-Dynamics Simulations

**Figure 4:**
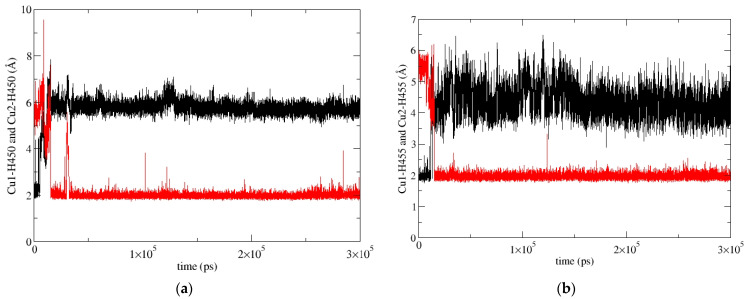
Exchange of Cu1 (red) and Cu2 (black) positions during the simulation SM1-1 replica of SM1 structure (hDPP III with Cu ions bound in mode 1), shown according to their distance from H450 (**a**) and H455 (**b**).

**Figure 5:**
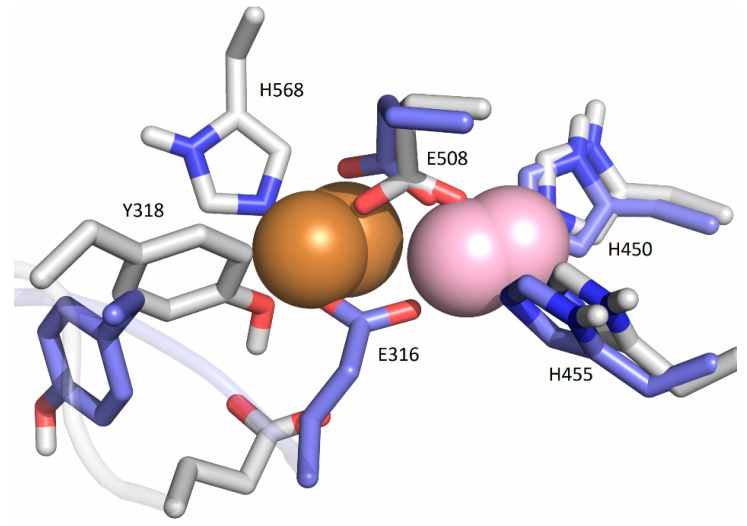
Coordination of Cu1 (light violet) with Cu2 (orange) in the SM1 structure: initial, represented as white sticks, and after the equilibration (blue sticks).

**Figure 6:**
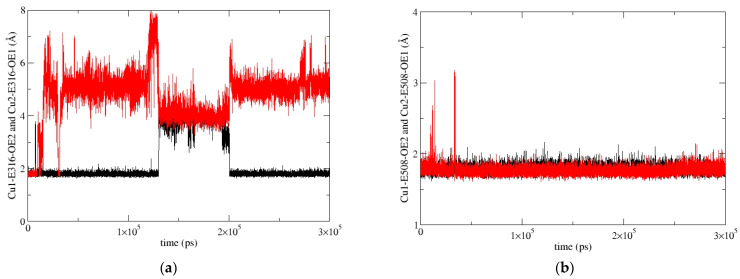
Coordination of Cu1 (red) and Cu2 (black) with E316 (**a**) and E508 (**b**) during MD simulations of the SM1-1 replica.

**Figure 7:**
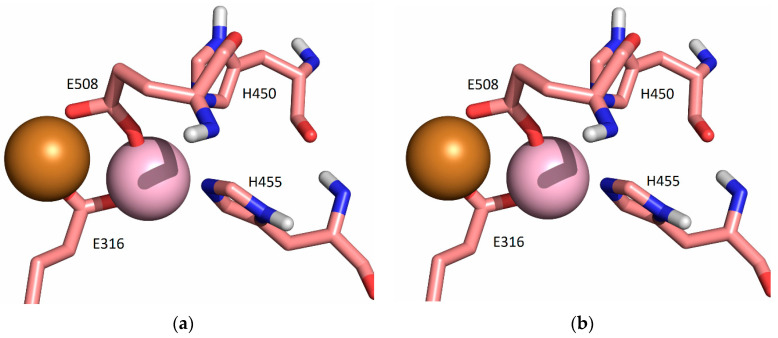
Coordination of Cu1 (light purple) and Cu2 (orange) with protein residues in the structure obtained after 3 ns (**a**) and 20 ns (**b**) of MD simulations of SM1 structure at room temperature, replica SM1-1 (the water molecules are not shown, for clarity).

In our previous work [19], we determined the main binding modes of the second zinc ion to hDPP III and traced the exchange of the ion in the additional, inhibitory, binding site with the catalytic ion. In this work, we investigated the binding of Cu ions to hDPP III. To clarify the relative stability of various di-copper hDPP III structures, MD simulations of the optimized structures of solvated di-metal protein (see Table S9 for some changes in geometry that occur during optimization) were performed (for details, see Section 4.2.2). In total, about 4 μs of MD simulations were performed with three different initial structures of di-copper hDPP III (Table S10; for the definitions of the initial structures, see Section 4.2.2 and Figure S10). The structure of the protein backbone remained stable during the MD simulations of all the structures (Figure S4), which was not true for the initial positions of the metal ions. The largest fluctuations of the Cu1 ion (Cu1 denotes the copper ion in the active center, and Cu2 denotes the second copper ion) were determined for the structures in which the copper ions were bound in mode 1 (SM1 structure) (Figure S5). In one of three simulated replicas of this structure, SM1-1, Cu1 and Cu2 exchanged positions (Figure 4; this was the only simulation in which the exchange of Cu ions occurred), and in one (SM1-3), the Cu1 left the catalytic site (see Figure S5). The expulsion of the Cu from the active site of the protein resulted in the decrease in the binding affinity of this copper ion for the enzyme, as approximated by the LIE energies (Table S11, P-Cu1 energy). The exchange of Cu ions occurred during the first 15 ns of the MD simulations (Figure 4). During the equilibration, the Y318 and H568 left the coordination sphere of the Cu2 and E316 ligated to both the Cu1 and the Cu2 (Figure 5), where they remained during the first 10 ns of the MD simulations (Figure 6). Over the next 5 ns, the Cu2 moved away from the E316, while the E508, which coordinated both metal ions throughout the simulation (Figure 6), rotated about 180° around the Cβ-Cγ bond (the dihedral Cα-Cβ-Cγ-Cδ changed from about 80° to −80°, Figure S6), and the Cu1 and Cu2 exchanged positions (Figure 7).

**Figure 8:**
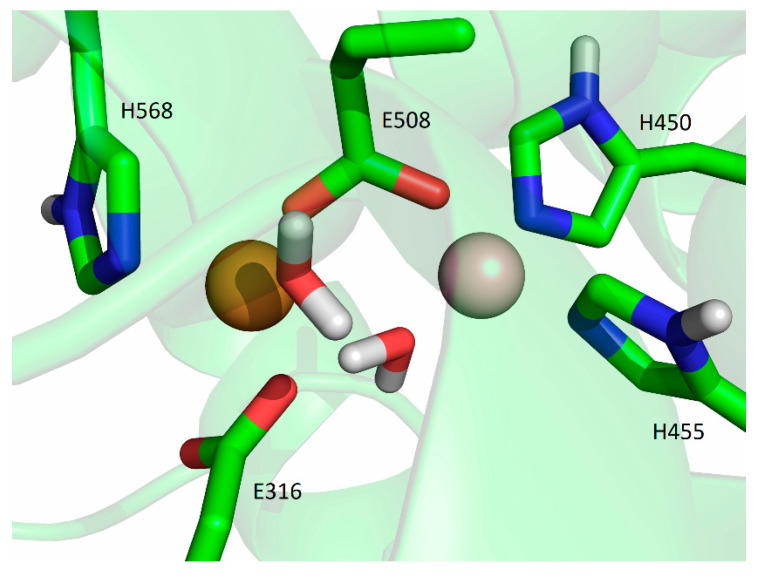
Coordination of Cu1 and Cu2 (both metal ions are represented as orange spheres) in the final structure obtained after 500 ns of MD simulations at room temperature, replica SM1-2.

During the simulation of the third replica of the DPP III structure with the Cu ions bound in mode 1 (SM1-2 simulation), the Cu1 constantly remained close to its initial position, and the H455 and E508 coordinated it throughout the simulation, but the H450 rotated about 100° around the Cβ-Cγ bond after about 70 ns (the dihedral Cα-Cβ-Cγ-Cδ changed from about −73° to 25°), and it was not coordinated with Cu1 during the following 250 ns. However, after about 330th ns of the MD simulation, it returned to its initial position and remained there until the end of the simulation (Figure 8 and Figure S5).

In the simulations of the structures with copper ions bound in mode 1′ and mode 2 (SM1′ and SM2 structure, respectively), the Cu1 remained close to its initial position throughout the simulations, ligated to the amino acid residues H450, H455, and E508 (Figure S7), and either to the hydroxide ion in the replica of the SM1′ structure or to the water molecule (in the replica of the S2 structure).

**Figure 9:**
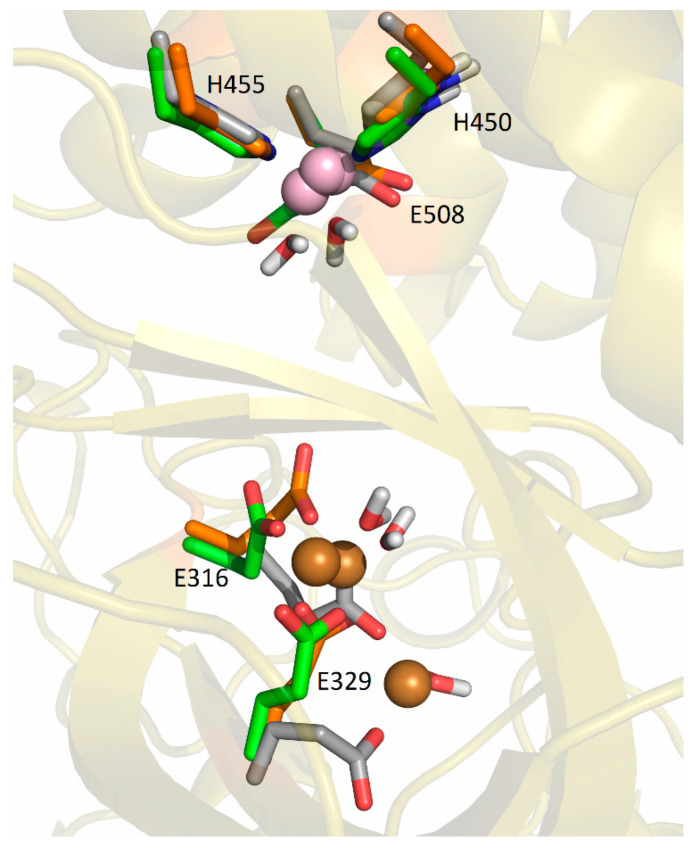
Coordination of Cu1 (light purple) and Cu2 (orange) in the final structures obtained by MD simulations of SM2 structure at room temperature, replicas SM2-1 (gray), SM2-2 (green), and SM2-3 (orange).

The fluctuations in Cu2 were largest in the SM2 structure. In all three replicas of this structure, it moved toward the lower domain in the direction of the entrance of the interdomain cleft, and it was accommodated between the E316 and the E329 (Figure 9, Figures S8 and S9). According to the LIE, PCu1-Cu2 energies, this is the most favorable way of binding the second copper ion (Table S11). In the simulations of the SM2 replica, the copper ions were mostly tetra-coordinated and, occasionally, penta-coordinated (it should be noted that the interaction of a metal ion with the carboxyl group of Glu is either monodentate, m, or bidentate, b). The Cu1 was coordinated by the H450, H455, E508^m^, and one water molecule (occasionally, two water molecules), and the Cu2 was coordinated by the E316^m^, E329^b^, and one water molecule in the SM2-1 and SM2-2 replicas. and by E316^m^, E329^m^, and two water molecules in the SM2-3 replica.

**Figure 10:**
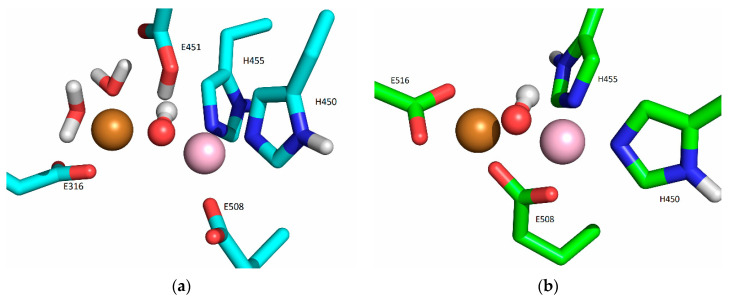
Coordination of Cu1 (light purple) and Cu2 (orange) with protein residues in the structure obtained after (**a**) 300 ns (S1′-1 replica, cyan), and (**b**) 200 ns (S1′-2 replica, green), of MD simulations of SM1′ structure at room temperature.

In the simulations of both replicas of the SM1′ structure, both copper ions remained near their original positions, as described above, the Cu1 was ligated with H450, H455, E508^m^, and the hydroxide and Cu2 was mostly ligated with E316^b^, E508^m^, and hydroxide in the SM1′-2 replica and with E316^m^, hydroxide, and two water molecules in the SM1′-1 replica (Figure 10 and Figure S9).

## 3. Discussion

The binding site of the catalytic Zn ion in DPP III is very similar to those in thermolysin (TML), carboxypeptidase A (CA), and anthrax lethal factor, i.e., in all of these, the zinc ion is coordinated by two histidines and a glutamate. In addition, all of these proteins can bind other divalent ions, such as Cu, Co, and Mn, excess metal ions inhibit their enzyme activity, and an inhibitory binding site has been identified [20,22,23]. It has also been shown that the binding of zinc and other metals in excess can lead to the inhibition of DPP III in humans, rats, and microorganisms [9,10,11]. All of these findings indicate that metal ions can bind not only at the catalytic site of DPP III, but also at an additional, so-called inhibitory binding site [19,20,21].

In this work, we focused on attempting to determine and identify the presence of an additional metal-binding site.

In the stopped-flow experiments, we showed that an excess of all the tested metals over the equimolar concentration to the protein leads to enzyme inhibition (Figure 1). The highest activity for all the metals was measured before or at the time point when the ratio of metal ions to protein molecules reached 1:1. The further addition of metal ions resulted in a similar decrease in activity for all the metal ions. We noted a slight difference between the activation of the protein by the zinc and the other metals: for Zn, the activity increased until the ratio of Zn to protein reached 1:1, and then decreased. For the other metal ions, the activity was mostly constant (maximum) up to the metal-to-protein ratio of 1:1, with Cu^2+^, an exception, reaching a peak at a Cu-to-protein ratio of 0.1.

The *K*_d_ values for the binding of the metal ions in the active site were measured fluorimetrically, as previously described [25,26,27]. Our results are in good agreement with previously published reports [11,28], with the greatest affinity for Cu^2+^ being picomolar and an order of magnitude higher than for the Zn^2+^, and three orders of magnitude higher than for Co^2+^, which is nanomolar.

The ICP-MS experiments gave clear and reproducible results. The method required the use of an ammonium-acetate buffer. When exposed to six molar equivalents of metal ions, the protein molecules retained 1.1 ions of zinc, 2.0 ions of copper, 0.3 ions of cobalt, and no manganese after the washing step. The presence of two copper ions per protein molecule indicated the presence of a secondary metal-binding site. Interestingly, only one zinc ion was bound. This seems to indicate the preference of the secondary site for copper or a kinetically more labile binding of zinc in the inhibitory site (leading to an exchange with the ion bound to the active site, which has already been suggested by molecular simulations [19]). After the washing step, no manganese remained bound to the enzyme, suggesting that manganese may bind only weakly. Surprisingly, the cobalt bound only a fraction of the protein molecules (30%). Previously, cobalt was shown to activate DPP III [9,11], presumably by binding in the catalytic metal-binding site. Interestingly, when the proteins were supplemented with manganese and cobalt, we were able to detect a considerable amount of zinc in the samples—between 20% and 50%. However, the source of this zinc could not be determined because all the samples were treated with the same procedure and no additional zinc was found in the apo and native protein samples. The simplest explanation for this finding could be that minute amounts of the chelators DPA and EDTA remained in the treated samples and were saturated by the addition of excess metals, allowing the minimal amounts of zinc ions present in the solutions to bind to the protein [29].

Another problem we encountered was the difference in reactivity between the native and apoproteins. The enzyme activity was greatly reduced in the apoprotein compared to the native protein. However, it could not be restored by the reconstitution of the holoprotein. We believe that, in order to successfully and completely remove metal ions, we overtreated the protein, rendering it mostly inactive. On the other hand, the native protein responded in a predictable and reproducible manner to the changes in metal-ion concentration. In the ICP-MS experiments, no differences were observed between the native and apo proteins. Our results indicate that the native protein is de facto an apoprotein, since the metal-ion content of the protein was very low (up to 10%, Table 1), and the stopped-flow experiments showed that the enzyme activity reached its maximum at equimolar concentrations of the metal ion. This suggests that recombinant protein production in *E. coli*, at least according to the protocol we used, produces a protein that does not contain sufficient amounts of metal-ion cofactors.

The results of the ICP-MS seemed to confirm our suspicion that there is an additional metal-binding site in hDPP III molecules. Since this method could only provide the stoichiometric relationship, we performed calorimetric experiments to obtain thermodynamic data on protein–metal complex formation. The ITC measurements revealed that Co^2+^ binds to only one binding site on hDPP III, presumably the active site. We were able to determine the *K*_d_ value and compare it with the fluorimetrically determined value, which resulted in a very good agreement between the two methods. The stoichiometric values for the Zn^2+^ and Cu^2+^ (*n* > 1) indicated the binding of at least two metal-ion-binding sites per protein molecule, which was further supported by the weak but measurable binding of Mn^2+^ to two binding sites (with non-integer stoichiometric values). The binding of the Zn^2+^ was reproducible in direct and reverse titration, with the largest difference in the stoichiometry, measured as *n* = 1.5 in the direct and *n* = 2.5 in the reverse titration. The partial exothermic-to-endothermic transition in the ITC curves also suggests the presence of at least two separate binding sites [30], with the low-affinity binding site directly measured [31]. The results of the immunoassays (see Supplement) suggest that the residual His-tag of the TEV protease, by binding two metal ions [32], may have increased the measured metal-ion content in the reverse titration by up to 20%, theoretically giving 2.4 metal ions per protein molecule, which is very close to the measured value in the reverse titration. The interaction we measured was used to determine the *K*_d_ value, which was in the order of 10^−7^ M. A similar, submicromolar affinity has already been measured for the lower-affinity binding site of zinc in β-lactamases [33,34].

For the Cu^2+^, the reverse titration did not confirm the direct titration, possibly due to interfering reactions with the buffer or the His-tag. Therefore, we did not consider these data as reliable. However, the results obtained for the binding of the Cu^2+^ to mutants E508D and E316A H568Y (Table S4) and the change in *K*_d_ suggest that the interaction measured by direct titration occurred with the protein molecule, in the vicinity of the catalytic-ion-binding site. We speculated that in the direct titration, we were measuring the binding of the Cu^2+^ to the same additional site as the Zn^2+^. This speculation led us to a *K*_d_ value of 10^−8^ M for this binding interaction, showing a slight preference for Cu^2+^ over Zn^2+^ for the additional binding site.

In the crystal structures of the metal-inhibited proteins TML and CPA, the second Zn ion is located near the active Zn ion, with the hydroxide ion bridging these two ions. In TML, the inhibitory Zn ion is additionally coordinated by His, Glu, and Tyr residues, whereas in CPA, it is bound to only one protein residue, Glu. In our previous study [19], we found that the inhibitory Zn ion binds preferentially to E508 (which bridges two metal ions), E316, and, occasionally to H568 or Y318. In the simulations, we found that the zinc in the catalytic region moved toward the entrance of the interdomain gap in the presence of the second zinc ion at the inhibitory binding site. At the same time, the metal ion from the inhibitory binding site moved to the catalytic center.

In 2016, Lo et al. showed that the replacement of the cognate Zn ions with the Cu ions in the LF increased the enzymatic activity. The fluorimetric measurements and QM calculations performed in this work showed that Cu binds with higher affinity to DPP III than Zn, and the enzymatic activity of Cu–DPP III is lower than that of Zn–DPP III. The latter observation is consistent with the results of the ITC measurements (through which we measured the binding of Zn and Cu at the additional, inhibitory binding site) and the results of the MD simulations, which predicted the lower mobility of the Cu bound in the inhibitory binding site compared with the Zn. It appears that zinc ions bound to the catalytic site and the inhibitory site are exchanged more frequently than copper ions; the zinc ions ejected from the active site rapidly leave the protein after exchange, while copper ions remain nearby.

The preference of DPP III for zinc is well established, as measured in native and recombinant proteins [35,36]. In general, our data suggest that the binding of transition metals to the hDPP III protein follows the universal Irving–Williams series of complex stabilities [37], Mn^2+^ < Co^2+^ << Cu^2+^ > Zn^2+^. The correct metallation is achieved through the cellular regulation of available amounts of metal ions [38].

In addition to the additional binding site suggested by the equivalence of the active sites of TML, CPA, and DPP III, which is located between the lower and upper domains of DPP III, the MD simulations showed that the Cu ion can bind with high affinity to the lower domain of DPP III, where it is bound to E316, E329, and a water molecule. The calorimetric measurements revealed different thermodynamic signature plots for the binding of the metal ions to DPP III. While the binding of copper ions is enthalpically driven, the binding of Zn and Co ions is entropically driven. On the other hand, the binding of the manganese ion in the active site is enthalpically controlled, while the binding to the additional site is entropically controlled. In general, different contributions to the Gibbs energy in enthalpy and entropy correspond to different binding modes [39]. There are several potential reasons for these differences, one of which is the difference in affinity of the metal ions for the buffer molecules, with Cu^2+^ shown to interact more intensely with the buffer components than other metal ions [40]. The other potential reason for this difference is the high affinity of Cu^2+^ for the binding site in the lower domain of DPP III, which seems to make the protein more rigid than the binding of the second metal near the catalytic site. The residues E316, Y318, E329, E508, and H568, all comprising the additional metal binding site(s), are conserved among DPPIIIs in eukaryotes and prokaryotes [17], suggesting a possible common mechanism of enzyme-activity regulation in this protease family.

## 4. Materials and Methods

### 4.1. Experimental Methods

#### 4.1.1. Chemicals

The purified protein sample was estimated to be 90% pure, based on SDS-PAGE gels. Tris buffer, sodium cacodylate buffer, LB broth, kanamycin, isopropyl-ß-D-thiogalacto-pyranoside (IPTG), imidazole, sodium dodecylsulphate (SDS), NaCl, acrylamide/bisacrylamide solution 29:1, (30% *w*/*v*), N,N,N′,N′-tetramethylethylenediamine (TEMED), ammonium peroxide, Rotigarose His/Ni beads, methanol, glycerol, glycine, sodium chloride, powdered milk, and acetic acid were acquired from Carl Roth (Karlsruhe, Germany). Amonium-acetate buffer, ammonium persulphate (APS), β-mercaptoethanol, Tween 20, bromphenol blue, MOPS buffer, and diphenylamine (DPA) were acquired from Sigma-Aldrich (St. Louis, MO, USA). The Arg_2_-2-naphthylamide (Arg-Arg–2NA) was produced by Bachem (Bubendorf, Switzerland); 2-naphthylamine (2-NA), 2-mercaptoethanol, and DNase I from bovine pancreas were produced by Merck (Darmstadt, Germany). For visualization of SDS-PAGE gels, we used PhastGel Blue R tablets (Pharmacia, Uppsala, Sweden). The EDTA was obtained from Kemika (Zagreb, Croatia), and metal (zinc, copper, cobalt, manganese) standard nitrate solutions were produced by Merck (Darmstadt, Germany).

#### 4.1.2. General

The human dipeptidyl-peptidase III (hDPP III) was expressed in *E. coli* strain BL21-CodonPlus(DE3)-RIL+ and purified by Ni-NTA affinity chromatography and fast protein liquid chromatography (FPLC). Protein concentrations were determined using microvolume spectrometer BioDrop (Biochrom, Cambridge, UK) by measuring protein A_280_ (absorbance at 280 nm), adjusted by the mass-extinction coefficient. All solutions were prepared using mQ ultrapure water. Further processing is described in detail in the following sections.

#### 4.1.3. Bacterial Transformation and Protein Expression

For the purpose of protein expression, *E. coli* strain BL21-CodonPlus (DE3)-RIL+ (Stratagene, San Diego, CA, USA) was transformed with pET28MHL plasmids containing the gene for hDPP III with a removable His-tag (original plasmid was a kind gift from Karl Gruber). Transformants were selected on kanamycin plates and grown in overnight bacterial cultures in liquid LB medium supplemented with kanamycin, at a final mass concentration of 100 μg mL^−1^, at 37 °C and 250 rpm. These cultures were used to inoculate an expression culture of 500 mL in medium of the same composition and under the same conditions. Expression cultures were grown to an optical density at 600 nm (OD_600_) ~ 0.6. After cooling to 18 °C for 30 min, protein expression was induced by the addition of IPTG at a final concentration of 0.25 mM. Expression was continued for 20 h at 18 °C. By centrifugation at 5500 rpm for 20 min, the bacterial cells were pelleted and stored at −20 °C until purification.

#### 4.1.4. Site-Directed Mutagenesis

Variants with altered amino acid sequences were prepared using QuikChange II XL Site-Directed Mutagenesis Kit (Agilent), following the instructions of the manufacturer. The sequences of the mutagenic primers are listed here, with altered bases in lowercase and altered codons underlined:
hDPP III_H455Y_F CACGAGCTGCTGGGTtAtGGCTCCGGCAAACTGhDPP III_H455Y_R CAGTTTGCCGGAGCCaTaACCCAGCAGCTCGTGhDPP III_H568Y_F CAACTGGCGCCAAGCCtAtATGCAGGCCCGTTTCGhDPP III_H568Y_R CGAAACGGGCCTGCATaTaGGCTTGGCGCCAGTTGhDPP III_E316A_F GTCTTACATTGGTTTCATCGcgTCTTACCGTGATCCTTTCGhDPP III_E316A_R CGAAAGGATCACGGTAAGAcgCGATGAAACCAATGTAAGAChDPP III_E508D_F CGAGCTCCTACGAGGAtTGTCGTGCAGAGTCTGhDPP III_E508D_R CAGACTCTGCACGACAaTCCTCGTAGGAGCTCG


Mutants’ full-length gene sequence was determined at Macrogen Europe. Proteins were expressed and purified using the same protocol as the wild type.

#### 4.1.5. Immunoassays/Western Blot

We performed Western-blot assays of protein samples used for stopped-flow, ICP-MS, and ITC measurements to confirm that His-tag removal from hDPP III was performed successfully. One to two micrograms of protein were loaded to a 12% gel, and SDS-PAGE and transfer to PVDF membrane was performed according to Laemmli and Towbin [41,42], using Bio-Rad Mini-Protean Tetra Cell and Mini Trans-Blot systems (Bio-Rad, Hercules, CA, USA). Membrane was stained using amido black and blocked using blocking buffer (5% (*w*/*v*) powdered milk in Tris-HCl-buffered saline solution with 0.1% (*v*/*v*) Tween 20). For detection, we used 1:5000 dilution of mouse Profinia anti-His (Bio-Rad 620-0203) as primary antibody and 1:20,000 dilution of goat anti-mouse IgG (H+L) HRP-conjugate (Proteintech SA00001-1, Proteintech, Rosemont, IL, USA) as secondary antibody. Antibodies were diluted in blocking buffer. The chemiluminescence was produced using Amersham ECL Prime Western Blotting Detection Reagent (Cytiva, Marlborough, MA, UASA) and detected on Alliance Q9 mini (Uvitec, Cambridge, UK), with exposition times from 30 s to 5 min. To quantify detected bands, we used an internal calibration curve with hDPP III and TEV protease, and analyzed blots using ImageJ (Bethesda, MD, USA).

#### 4.1.6. Protein Purification

Bacterial pellets were resuspended at 4 °C in lysis buffer (50 mM Tris-HCl, 300 mM NaCl, pH 8.0) with 1 mg mL^−1^ lysozyme and sonicated to break up the bacterial cells. Lysates were centrifuged at 14,500× *g* to separate soluble proteins from cell-residue precipitates. The soluble fraction above the precipitate was filtered through a 0.45-micrometer-diameter pore filter before application to a Ni-NTA column. The volume of the column was 6 mL for about 50 mL of bacterial cell lysate. The lysate was applied to a column (equilibrated in lysis buffer) at a flow rate of 0.5 mL min^−1^, followed by elution at a flow rate of 1.0 mL min^−1^ wash buffer (lysis buffer with the addition of 20 mM imidazole), and proteins were finally eluted in buffer with 300 mM imidazole (50 mM Tris-HCl, 300 mM NaCl, 300 mM imidazole, pH 8.0). Protein concentration was determined by BioDrop, by measuring protein absorbance at 280 nm. The His-tag used for affinity chromatography was removed by TEV protease. Further purification was performed by gel-filtration chromatography on a FPLC Åkta protein-chromatography system (Pharmacia, NJ, USA), using a Superdex S200 16/60 column. The collected protein fractions were analyzed by SDS-PAGE. The gels were stained with Coomassie Brilliant Blue R-250. Protein aliquots were stored at −80 °C.

#### 4.1.7. Preparation of Apo hDPP III

Metal-free apoprotein was prepared from wild-type hDPP III. The Wt hDPP III was dialyzed in dialysis vials or dialysis tubing (10 MWCO) for 24 h at room temperature in pH 7.4 buffer containing 25 mM ammonium acetate, 10 mM ethylenediaminetetraacetic acid (EDTA), and 1 mM dipicolinic acid (DPA). The removal of excess EDTA and DPA was performed through sequential washing with 25 mM ammonium acetate pH 7.4, and the concentration of the protein sample was performed through filtration on Amicon Ultra 15 (30 MWCO) columns (Merck).

#### 4.1.8. Preparation of Holoprotein

Apoprotein (approximately 6 μM protein) was incubated for 1 h with 36 μM solutions of metal nitrites Zn^2+^, Cu^2+^, Co^2+^, and Mn^2+^ in 25 mM ammonium acetate buffer, pH 7.4, after which the excess metal was immediately removed by washing with the same ammonium acetate buffer on an Amicon Ultra 15 (30 K) column, using centrifugation. Final protein-sample concentrations were determined as described above and diluted to a mass concentration of 0.05 mg mL^−1^ with the washing buffer.

#### 4.1.9. Determination of Zinc, Copper, Cobalt, and Manganese by Stopped-Flow Spectrophotometry

The exchange of zinc and metals was monitored by fluorescence spectrophotometry on a stopped-flow instrument (SX20 stopped-flow spectrometer Applied Photophysics, Beverly, MA, USA) using two syringes. One syringe was filled with WT protein concentration 10 nM and metal-nitrate solution from a basic solution with a concentration of 15 mM and diluted in 40 mM Tris-HCl buffer, pH 7.5. The second syringe was filled with substrate (200 µM Arg-Arg-2NA). The reference reaction contained 200 nM protein and 200 µM substrate. All measurements were performed at room temperature at a single wavelength, 332 nm. Pro-Data Viewer analysis software v4.2.5, supplied by the manufacturer, was used for data analysis.

#### 4.1.10. Determination of Dissociation Constant—Fluorimetric Measurements

The dissociation constant of proteins with Zn, Cu, and Co dications was estimated by assessing the activity of the enzyme with metal nitrates in 20 mM Tris-HCl buffer, pH 7.4, with DPA serving as the chelator. Experiments were performed on a Cary Eclipse Fluorescence Spectrophotometer (Agilent Technology, Santa Clara, CA, USA), which measures the release of β-naphthylamine upon cleavage of the synthetic substrate Arg-Arg-2NA. All experiments were performed under the same conditions, at room temperature, at an extinction wavelength of 332 nm and an emission wavelength of 420 nm, for 60 s of reaction time. The experiment was performed with aliquots of 10 nM enzyme hDPP III, 0.8 mM substrate Arg-Arg-2NA, 10 mM DPA chelator, and standard metal-nitrate solutions.

For the calculation of the free-metal-ion concentration, the stability constants of the metal–DPA complex found in the literature were used, specifically at pH 7.4 for Zn^2+^ (β1 = 10^7^, β2 = 10^13^) and Cu^2+^ (β1 = 10^10^, β2 = 1.99 × 10^16^) [11], as well as for Co^2+^ (β1 = 10^7^, β2 = 3.2 × 10^12^) [27]. The dissociation constant *K*_d_ was determined in GraphPad Prism 5.04/d data-analysis software, using a sigmoidal 4PL non-linear regression. Thus obtained *K*_d_ values were corrected for metal-ion–buffer interactions using Equations (3) and (4) [43], and *K*_MB_ values obtained from [43] as log*K*_MB_ = 2.27 for Zn^2+^, 1.47 for Cu^2+^, and 1.73 for Co^2+^ ions at pH 7.4.

(3) $$K_{a}=K_{a,app}\times Q_{MB}$$

(4) $$Q_{MB}=1+K_{MB}\times [buffer]$$

#### 4.1.11. Analysis of Metal-Ion Content by ICP-MS

According to the described protocol for the preparation of apo and holoproteins, the metal content was detected using the triple-quadrupole Agilent 8800 (Agilent Technologies, Tokyo, Japan) ICP-MS instrument. Prior to analysis, samples were diluted 4-fold with an alkaline diluent solution containing 0.7 mM NH_3_, 0.01 mM EDTA, 0.07% (*v*/*v*) TX-100, and 3 µg L^−1^ of internal standards (Ge, Rh, Tb, Lu, and Ir) (SCP Science, Baie D’Urfé, QC, Canada).

Matrix-matched calibration was used for the quantification of Zn, Cu, Co, and Mn concentrations (multielement calibration curve made from single element standards from SCP Science). A calibration curve for S was prepared separately by diluting working S standards (Inorganic Ventures, Christiansburg, VA, USA) with the diluent solution.

The accuracies of measurements were checked using commercially available reference materials: ClinChek® Serum Controls (Level I and II) (Recipe, Munich, Germany) and SeronormTM Serum (Level I and II) (Sero AS, Billingstad, Norway) prepared by 20-fold dilution of reconstituted freeze-dried reference material with diluent solution. Analyzed elements in the referent biological samples were within ±9% of the certified values.

#### 4.1.12. Isothermal Titration Calorimetry (ITC)

Isothermal titration calorimetry (ITC) experiments were performed on a Malvern PEAQ-ITC microcalorimeter (MicroCal, Inc., Northampton, MA, USA). Experiments were performed in 50 mM sodium cacodylate, pH 7.4, and 50 mM MOPS-NaOH buffer, pH 7.4 at 25 °C. All standard solutions of metal ions were nitrates. Protein was dialyzed and metal salts dissolved in the same buffer solution, which was used for further dilutions. For direct titration, protein solution (20–40 μM) was in the cell (200 μL) and metal solution (200–400 μM) was in the syringe (40 μL). For reverse titrations, protein solution was concentrated using Amicon concentration devices (120–500 μM) and loaded in the syringe, while metal solution (10–60 μM) was in the cell. All experiments were performed under the same conditions of temperature 25 °C, reference power 30.0 μW, high feedback, stirring speed 500 rpm, spacing 150 s, and initial delay 60 s to allow equilibration. Experiments to correct for heat of dilution (buffer–buffer, peptide–buffer, buffer–protein) were performed for all experiments. During analyses, all control experiments were subtracted from every binding experiment. The MicroCal PEAQ-ITC analysis software v1.30, supplied by the manufacturer, was used for data analysis. One set of sites and two sets of sites fitting models were used to find the best fit for experimental data. All parameters are presented as average value and standard deviation of at least two, and mostly three or four measurements. Apparent *K*_d_ values were corrected for metal–buffer interactions [43] using Equations (3) and (4). The *K*_MB_ values were obtained from previous works [43,44,45], and those not measured at pH 7.4 were recalculated using buffer p*K*_a_ values, as described previously [44].

### 4.2. Computational Methods

#### 4.2.1. Quantum Mechanical Calculations (QM)

The 3D structures of 14 different complexes, as well as composition models representing the active enzyme site of the enzyme hDPP III with different metal ions, Zn^2+^, Cu^2+^, Co^2+^, and Mn^2+^, were optimized by density functional theory (DFT) calculations in combination with the density-based solvation model (SMD) [46], as implemented in the program Gaussian 09. To allow comparison, all structures were also optimized in vacuum.

1. Model preparation

**Figure 11:**
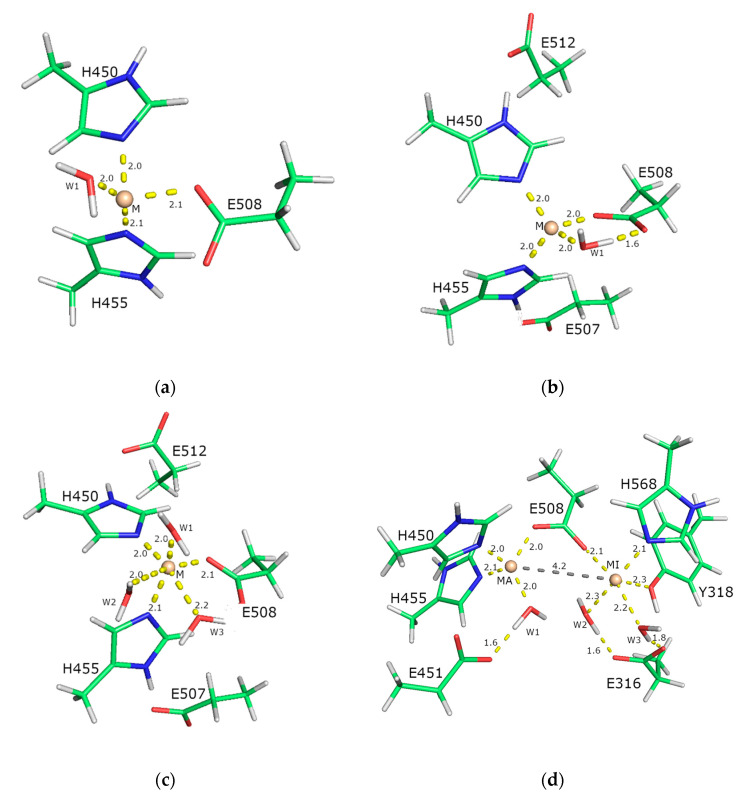
The models used in this study. Models of the catalytic-metal-ion-binding site in hDPP III are shown in figures (**a**–**c**), and the model of hDPP III with two bound metal ions is shown in figure (**d**). The smallest model 1 (**a**) comprises only the first coordination sphere of the metal ion (the metal ion coordinated with three amino acids and one water molecule) The models shown in (**b**,**c**) also include the amino acids from the second coordination sphere, i.e., five amino acids in total, and they differ only in the number of water molecules. Model 2 (**b**) has one and model 3 (**c**) has three water molecules. In all structures (**a**–**c**), the positions of the metal ions M^2+^ (M represents ions Zn^2+^, Cu^2+^, Co^2+^, and Mn^2+^) is indicated by the sphere, and the amino acids and water molecules are shown as sticks. In bimetallic model 4 (**d**), the catalytically active metal ion is indicated by MA and the inhibitory ion by MI. Distances are in Å.

The closed structure of the ligand-free hDPP III (PDB_id 5EGY) served as a template. In terms of complexity, four different models were investigated: three mononuclear binding sites and one binuclear binding site. The simplest model contained only the metal ion, one water molecule, and the side chains of the amino acid residues of the first coordination sphere. The more complex model also contained the amino acid residues of the second coordination sphere, and the most complex model additionally contained two further water molecules (see Figure 11). In the experimental structure, the zinc ion is coordinated by H450, H455, E508, and a water molecule. The E507 and E512 belong to the second coordination shell of the metal ion and stabilize the H450 and H455 from the first coordination shell. Thus, the simplest model (model 1, Figure 11a) involves a metal ion in the active site coordinated by amino acids H450, H455, and E508, as well as a water molecule. The other two models include one metal in the active site, amino acids H450, H455, E507, E508, and E512, and one (model 2, Figure 11b) or three (model 3, Figure 11c) water molecules. The binuclear binding site (model 4, Figure 11d) was constructed from the QM part of the optimized QM/MM structure (Figure S1) in our previous work [19] and included amino acid residues coordinating metal ions, the catalytic (H450, H455, and E508) and inhibitory metal ion (E508, H568, and Y318, E316), and E451. The carboxyl group of E508 bridged metal ions.

2. Details of QM calculations

The geometry of the constructed models was optimized using the DFT method in combination with density-continuum-based solvation model (SMD) with a dielectric constant (ε) of 4, which was used to simulate the protein environment of the metal ions. The use of DFT calculations has been shown to reliably reproduce geometric and biological systems, as well as the thermodynamic data associated with their transformation [47,48,49]. The calculations were carried out using the Gaussian 09 suite of programs [50], employing the unrestricted B3LYP functional. The B3LYP uses the non-local correlation functional expressed by Lee, Yang, and Parr [51] and a hybrid three-parameter-exchange functional devised by Becke [52]. All calculations were performed with a double-ζ basis set 6-31G(3d,p), employing the unrestricted B3LYP functional. In this way, the electronic energies, Eel, of the optimized systems were obtained. According to previous studies, the B3LYP/6-31G(3d,p) level of theory represents a good balance between accuracy and computational resources for obtaining the necessary structural and thermodynamic parameters for the systems representing biological systems with metal dications, such as Zn^2+^, Cu^2+^, Co^2+^, and Mn^2+^ and metal-ion hydration [48,53,54,55,56,57]. Each metal complex was optimized in the gas phase, with the methyl groups capping the models of constrained amino acid residues.

Frequency calculations were performed at the same 3LYP/6-31G(3d,p) level of theory in order to confirm that the minimized structure represented a true local minimum on the potential energy surface of the respective metal complex. No imaginary frequencies were found for any of the structures studied.

The differences in Eel, Eth, and S between the metal in the active enzyme site and in the solvent in Equation (5) were used to evaluate the metal-exchange Gibbs energy in the gas phase, ΔG, at T = 298.15 K and 1 atm, according to:

ΔG = ΔEel + ΔEth − TΔS(5)

The relative affinity of different metals towards the DPP III active site was calculated using Equation (6) and, for systems in vacuum, Equation (7), where M1 is cognate Zn^2+^ and M2 is either Cu^2+^, Co^2+^, or Mn^2+^.

ΔG = ΔGε_1_([M1 + P]) − ΔGε_1_([M2 + P]) − ΔGε_1_([M1 + water]) + ΔGε_1_([M2 + water]) (6)

ΔG = ΔGε_3_,ε_2_([M1 + P]) − ΔGε_3_,ε_2_([M2 + P]) − ΔGε_3_,ε_2_([M1 + water]) + ΔGε_2_([M2 + water]) (7)

where ε_1_ = 4, ε_2_ = 78, and ε_3_ = 1.

The PyMOL molecular graphics system was used to generate the molecular graphics images (PyMOL version 1.5.x Molecular Graphics System, Schrödinger, LLC, New York, NY, USA).

#### 4.2.2. Molecular-Dynamics Simulations (MD)

DPP III Structures With Di-Copper Sites and System Preparations.

By analogy with the di-zinc structures of DPP III, we have considered three modes of binding of Cu ions to DPP III. In all of them, Cu1 is located at the position of the catalytic metal ion and is coordinated to H450, H455, and E508. In the DPP III structure with metal ions bound in so-called mode 1 (structure SM1) Cu2 is coordinated to Y318, H568, and E508 which bridges Cu1 and Cu2, while in mode 2 (structure SM2), Cu2 is instead to Y318, coordinated to E316. In mode 1′ (structure SM1′) Cu ions are coordinated with the same amino acid residues as in mode 1, but Cu1 and Cu2 are bridged by OH- in addition to E508 (Figure S10).

1. System Preparations

For molecular modeling, the protonation of the charged residues and histidines was adjusted to a pH of about 7.5, as expected under physiological conditions. Thus, the arginine and lysine residues were positively charged in our models, whereas the glutamate and aspartate residues were negatively charged, with the exception of E451, which was neutral according to our previous results on di-zinc DPP III. The histidines were neutral and the position of the hydrogen atoms on the imidazole ring was chosen according to their ability to form hydrogen bonds with neighboring amino acid residues or to coordinate a metal ion. The protein was parameterized using the ff19SB force field [58] and standard unbound parameters for Cu^+^ [59] available within AMBER suit of programs. The system was solvated using the truncated octahedron of TIP3P water molecules [60].

The distance of the molecular surface from the box was at least 11 Å. Na^+^ ions were added to achieve electroneutrality. All MD simulations were performed using the AMBER20 suite of programs [61].

2. Classical MD simulations

Prior to the productive MD simulations, the systems were optimized in three cycles with different constraints. In the first cycle (1500 minimization steps), aimed at relaxing the solvent molecules, the protein and zinc ion were constrained by a harmonic potential with a force constant of 32 kcal mol^−1^ Å^−1^. In the second cycle (3500 minimization steps), only the protein backbone was constrained with a force constant of 12 kcal mol^−1^ Å^−1^, while the entire system was minimized in the third cycle (2500 minimization steps) without additional constraints. The systems were heated in three steps from 0 to 300 K, from 0–100 K, from 100–200 K, and from 200–300 K during 50 ps. This was followed by a 3 ns density equilibration at 300 K. A time step of 0.5 fs was used for the heating simulations and 1 fs for the equilibration simulations.

In the productive MD simulations we used the algorithm SHAKE [62] and a time step of 2 fs. During heating, the NVT ensemble was used, while equilibration and production MDs were performed with the NPT ensemble, with a cutoff value of 11 Å. During the simulations, the temperature was controlled using the Langevin thermostat [63] with a time interval between temperature rescaling of 0.5 ps during heating and density equilibration and of 1 ps during MD simulations. Pressure was controlled using the Berendsen barostat [64] with a relaxation time of 1.0 ps. A total of 4 μs of productive classical MD simulations were performed for various di-copper DPP III structures.

3. Data analysis

Calculations of geometry parameters (RMSD, Rgyration, and RMSF metal-ion coordination) and analysis of linear interaction energies (LIE) were performed using the cpptraj module [65] of the AmberTools20 program package. Figures were generated using PyMOL (PyMOL Molecular Graphics System, version 1.5.0.4, Schrödinger LLC, New York, NY, USA).

## 5. Conclusions

In this work, we demonstrated the binding of Zn^2+^ and Cu^2+^ metal ions to an additional metal-binding site of the hDPP III molecule. Under the conditions used for the ICP-MS experiments, two Cu^2+^ ions remained bound to the protein. Using ITC, the binding of Zn^2+^ to the additional binding site was confirmed in direct and reverse titrations, and the affinity of the interaction was quantified as a 10^−7^ M *K*_d_ value. The QM and molecular-mechanics calculations also revealed the existence of an additional, high-affinity binding site for copper and zinc ions near the catalytic-metal-ion-binding site. On the other hand, both the experimental (fluorescence) and the computational method (QM calculations) showed a higher affinity of the copper ion than the zinc ion for the active binding site.

Furthermore, the MD simulations showed that Cu and Zn ions can exchange their positions at the catalytic and additional binding sites, suggesting that when two zinc ions bind, one of them leaves the protein more frequently than when two Cu ions bind to hDPP III.

Thus, the additional (inhibitory) binding site was biochemically confirmed by experimental and computational methods; however, the physiological relevance of our results might be questioned. Overall, we conclude that we collected sufficient data to support our hypothesis that DPP III has an additional metal-ion-binding site, whose affinity for zinc is four orders of magnitude lower than that of the active site. The location of the active site is similar to that identified in other distantly related proteases, suggesting a common mechanism of regulation of enzyme activity by excess metal ions.

## Figures and Tables

**Table 1:** Number of metal ions relative to protein molecules (*N*) measured by inductively-coupled-plasma mass spectrometry using hDPP III holoenzymes. The results are shown as the average and standard deviation of two measurements.

hDPP III	*N* (Zn^2+^)	*N* (Cu^2+^)	*N* (Co^2+^)	*N* (Mn^2+^)
native	0.07 ± 0.04	0.01 ± 0.00	0.01 ± 0.00	0.00 ± 0.00
apo	0.02 ± 0.01	0.00 ± 0.00	0.02 ± 0.00	0.00 ± 0.00
apo + Zn^2+^	1.07 ± 0.16	0.02 ± 0.01	0.01 ± 0.01	0.01 ± 0.02
apo + Cu^2+^	0.05 ± 0.05	2.03 ± 0.09	0.02 ± 0.01	0.01 ± 0.01
apo + Co^2+^	0.37 ± 0.17	0.03 ± 0.03	0.33 ± 0.01	0.00 ± 0.00
apo + Mn^2+^	0.32 ± 0.03	0.04 ± 0.02	0.05 ± 0.04	0.03 ± 0.01

**Table 2:** Best-fit values of thermodynamic parameters determined for metal–protein interactions using ITC in 50 mM sodium cacodylate buffer at pH 7.4—apparent values from direct titrations (metal ions to protein). The results are shown as the average and standard deviation of three measurements.

	*n*_app_	*K*_d,app_/μM	Δ_r_*H*_app_/kJ mol^−1^	Δ_r_*G*_app_/kJ mol^−1^	−*T* Δ_r_*S*_app_/kJ mol^−1^	Supposed Binding Site
Zn^2+^	1.46 ± 0.07	1.0 ± 0.8	18 ± 2	−34 ± 1	−52 ± 1	additional
Cu^2+^	1.2 ± 0.1	2.4 ± 0.7	−49.8 ± 0.3	−32.2 ± 0.7	17.6 ± 0.5	additional
Co^2+^	0.63 ± 0.04	0.13 ± 0.02	39 ± 1	−36.7 ± 0.5	−78 ± 1	active
Mn^2+^ (1)	0.22 ± 0.01	0.23 ± 0.05	−42 ± 1	−38.0 ± 0.5	4 ± 2	active
Mn^2+^ (2)	0.44 ± 0.04	1.0 ± 0.1	47.0 ± 0.5	−34.3 ± 0.4	−81.2 ± 0.5	additional

**Table 3:** Dissociation constants for the binding of metal ions to DPP III, determined using ITC—apparent values were corrected for the interaction of metal ions with the buffer.

Metal Ion	Buffer	*K*_d,app_/M	log*K*_MB_	*Q*_MB_	*K*_d_/M	Binding Site
Zn^2+^	Na-cacodylate	9.6 × 10^−7^	2.14	8.0	1.2 × 10^−7^	additional
Zn^2+^	MOPS-NaOH	8.8 × 10^−6^	3.22	84	1.0 × 10^−7^	additional
Cu^2+^	MOPS-NaOH	5.5 × 10^−6^	3.8	313	1.8 × 10^−8^	additional
Co^2+^	Na-cacodylate	1.3 × 10^−7^	2.27	10.4	1.3 × 10^−8^	active

**Table 4:** The relative Gibbs free energies (compared to Zn^2+^) for non-native metals for the binding site of hDPP III. Calculations were performed according to Equation (1), using the three models with different levels of complexity (see above) to approximate the active protein site. (**A**) All calculations were performed with ε = 4. (**B**) The energy optimization in vacuum followed by a single-point energy calculation with ε = 78.

Metal Cation (M^2+^)	Relative Affinities/kcal mol^−1^		
(**A**)			
	Model 1	Model 2	Model 3
Cu^2+^	−5.65	0.63	−2.51
Co^2+^	3.77	4.39	−31.38
Mn^2+^	11.30	15.06	33.89
(**B**)			
Cu^2+^	−10.67	−4.39	2.51
Co^2+^	5.02	−1.26	9.41
Mn^2+^	12.55	13.18	25.1

## Data Availability

Data available on request.

## Supplementary Materials

The following supporting information can be downloaded at: https://www.mdpi.com/article/10.3390/ijms241612747/s1.
