# Supplementary material for: Identification of an Additional Metal-Binding Site in Human Dipeptidyl Peptidase III
Source: Int J Mol Sci. 2023 Aug 13;24(16):12747. doi: 10.3390/ijms241612747 (PMC10454320; doi:10.3390/ijms241612747)
Supplement: Supplementary file 1 [file ijms-24-12747-s001.zip › ijms-2528845-supplementary.pdf]

Supplementary Material

Identification of an Additional Metal Binding Site in Human Dipeptidyl Peptidase III

Antonia Matić, Filip Šupljika, Hrvoje Brkić, Jasna Jurasović, Zrinka Karačić and Sanja Tomić

Table of Contents

Supplementary Results ……………………………………………………………..………………... 1

Supplementary Tables……………………………………………………………………..……. 2 - 12

Supplementary Figures…………………………………………………………………………....13 - 22

Supplementary Results

Immunoassays

We used western blot to confirm that the His-tag used to purify hDPP III was successfully removed from the protein, as the affinity of the tag for transition metal ions could interfere with the metal ion content measurements. To cleave the His-tag, we used TEV protease, also labelled with the same affinity tag. The removal of the His-tag from hDPP III was confirmed using immunoassay, but we noticed some contamination with the TEV protease (Fig. S2). To quantify the remaining TEV protease, we used a calibrating curve with 10-200 ng TEV protease, imaged on the same membrane as the investigated sample (Fig. S3). The amount of contaminating protein was calculated from the calibrating curve and determined to be 67 ng of TEV protease in a sample of 1000 ng hDPP III. If both hDPP III and TEV protease bind two metal ions [1,2], our calculations suggest that the contamination could alter the expected metal content in the protein sample by maximum 20%, but not by an integer value. The procedure was performed for all samples, and yielded similar results.

References:

1. Tomić, A.; Brkić, H.; Matić, A.; Tomić, S. Unravelling the Inhibitory Zinc Ion Binding Site and the Metal Exchange Mechanism in Human DPP III. *Phys. Chem. Chem. Phys.* **2021**, *23*, 13267–13275, doi:10.1039/d1cp01302e.
2. Smolko, A.; Šupljika, F.; Martinčić, J.; Jajčanin-Jozić, N.; Grabar-Branilović, M.; Tomić, S.; Ludwig-Müller, J.; Piantanida, I.; Salopek-Sondi, B. The Role of Conserved Cys Residues in Brassica Rapa Auxin Amidohydrolase: Cys139 Is Crucial for the Enzyme Activity and Cys320 Regulates Enzyme Stability. *Phys. Chem. Chem. Phys.* **2016**, *18*, 8890–8900, doi:10.1039/c5cp06301a.

Supplementary Tables

Table S1. ITC results - reverse titration in sodium cacodylate buffer. Best-fit values of thermodynamic parameters determined for metal-protein interactions using ITC in 50 mM sodium cacodylate buffer pH 7.4 – apparent values from reverse titrations (protein to metal ions)

metal ion	n_{app}	$K_{d,app}$ / μM	$\Delta_r H_{app}$ / kJ mol^{-1}	$\Delta_r G_{app}$ / kJ mol^{-1}	$-T\,\Delta_r S_{app}$ / kJ mol^{-1}	supposed binding site
Zn^{2+}	2.5 ± 0.2	3.5 ± 2	15 ± 5	-31 ± 2	-46 ± 3	additional
Cu^{2+}	1.7 ± 0.1	1.0 ± 0.3	-24 ± 3	-34.4 ± 0.8	-11 ± 4	?
Co^{2+}	0.62 ± 0.04	0.7 ± 0.2	44.5 ± 0.9	-35.2 ± 0.6	-80 ± 2	active
Mn^{2+} (1)						n.d.
Mn^{2+} (2)	0.44 ± 0.01	0.95 ± 0.09	52 ± 2	-34.5 ± 0.2	-87 ± 1	additional

Table S2. ITC results - Zn^{2+} and Cu^{2+} in MOPS-NaOH buffer. Best-fit values of thermodynamic parameters determined for metal-protein interactions using ITC in 50 mM MOPS-NaOH buffer pH 7.4 – apparent values from direct and reverse titrations

metal ion	n_{app}	$K_{d,app}$ / µM	$\Delta_r H_{app}$ / kJ mol^{-1}	$\Delta_r G_{app}$ / kJ mol^{-1}	$-T\,\Delta_r S_{app}$ / kJ mol^{-1}	supposed binding site
direct						
Zn^{2+}	1.5 ± 0.3	9 ± 4	20 ± 7	-29 ± 2	-49 ± 6	additional
Cu^{2+}	0.8 ± 0.1	5 ± 1	-50 ± 6	-30.1 ± 0.7	20 ± 6	additional
reverse						
Zn^{2+}	2.6 ± 0.2	1.8 ± 0.8	6 ± 2	-33 ± 1	-39.7 ± 0.3	additional
Cu^{2+}		8 ± 5				?

Table S3. ITC results - mutants and Zn^{2+}. Best-fit values of thermodynamic parameters determined for Zn-protein interactions with hDPP III mutants using ITC in 50 mM sodium cacodylate buffer pH 7.4 – apparent values from direct titrations. Data for wild-type is given for comparison.

	n_{app}	$K_{d,app}$ / μM	$\Delta_r H_{app}$ / kJ mol^{-1}	$\Delta_r G_{app}$ / kJ mol^{-1}	$-T\,\Delta_r S_{app}$ / kJ mol^{-1}
wild-type	1.46 ± 0.07	1.0 ± 0.8	18 ± 2	-34 ± 2	-52 ± 1
E508D	1.3 ± 0.3	1.9 ± 0.3	19 ± 3	-32.7 ± 0.4	-52 ± 3
E316A H568Y	1.8 ± 0.1	3 ± 2	16 ± 5	-32 ± 2	-48 ± 3

Table S4. ITC results - mutants and Cu^{2+}. Best-fit values of thermodynamic parameters determined for Cu-protein interactions with hDPP III mutants using ITC in 50 mM sodium cacodylate buffer pH 7.4 – apparent values from direct titrations. Data for wild-type is given for comparison.

	n_{app}	$K_{d,app}$ / μM	$\Delta_r H_{app}$ / kJ mol^{-1}	$\Delta_r G_{app}$ / kJ mol^{-1}	$-T\,\Delta_r S_{app}$ / kJ mol^{-1}
wild-type	1.2 ± 0.1	2.4 ± 0.7	-49.8 ± 0.3	-32.2 ± 0.7	17.6 ± 0.5
E508D	0.7 ± 0.1	8 ± 5	-92 ± 12	-29 ± 1	63 ± 13
E316A H568Y	0.8 ± 0.5	14 ± 2	-88 ± 15	-27.7 ± 0.4	60 ± 16

Table S5. Selected distances (Å) in the DPP III structures model1 with metal ions (Zn, Cu, Co and Mn) before (I) and after energy optimization.

	Distance between the metal ion and the amino acid residues from the 1st coordination sphere (Å)				
Model1	I	Zn	Cu	Co	Mn
M-H450	2.0	2.0	2.0	2.0	2.1
M-H455	2.1	2.0	2.0	2.0	2.1
M-E508	2.1	2.1	2.0	2.0	2.1
M-w1	2.0	2.0	2.3	2.1	2.1
E508-w1	3.6	1.6	3.0	1.7	1.6

Table S6. Selected distances (Å) in the DPP III structures model2 with metal ions (Zn, Cu, Co and Mn) before (I) and after energy optimization.

	Distance between the metal ion and the amino acid residues from the 1st coordination sphere (Å)				
Model2	I	Zn	Cu	Co	Mn
M-H450	2.0	2.0	2.0	2.0	2.1
M-H455	2.0	2.0	2.0	2.0	2.1
M-E508	2.0	2.0	2.1	2.0	2.1
M-w1	2.1	2.0	2.3	2.0	2.1
E508-w1	1.6	1.6	2.9	1.6	1.6

Table S7. Selected distances (Å) in the DPP III structures model3 with metal ions (Zn, Cu, Co and Mn) before (I) and after energy optimization.

	Distance between the metal ion and the amino acid residues from the 1st coordination sphere (Å)				
Model3	I	Zn	Cu	Co	Mn
M-H450	2.0	2.0	2.0	2.1	2.1
M-H455	2.1	2.0	2.0	2.1	2.1
M-E508	2.1	2.0	2.3	2.1	2.2
M-w1	2.0	3.4	2.1	2.3	2.3
M-w2	2.5	3.1	2.8	2.2	2.2
M-w3	2.2	2.1	2.0	2.3	2.3
E508-w1	3.6	4.5	3.5	1.8	1.6
E508-w3	1.2	1.6	1.5	1.8	1.6

Table S8. Selected distances (Å) in the DPP III structures model4 with two metal ions (either two Zn or two Cu) before (I) and after energy optimization with either Zn or Cu.

	Distance (Å)		
Model4	I (MA and MI)	Zn (MA and MI)	Cu (MA and MI)
MA-MI	4.2	5.5	5.2
MA-H450	2.0	2.1	2.0
MA-H455	2.1	2.1	2.0
MA-E508	2.0	2.0	1.9
MI-E508	2.1	3.6	3.8
MI-H568	2.1	2.0	1.9
MI-Y318	2.3	2.1	2.2
MA-w1	2.0	1.9	2.1
MI-w2	2.3	2.0	2.0
MI-w3	2.2	1.9	1.8
E451-w1	1.6	2.6	1.7
E316-w2	1.6	1.8	1.8
E316-w3	1.8	2.6	1.0

Table S9. Selected distances (Å) in the DPP III structures with two Cu ions before (I) and after energy optimization (for details of energy optimization protocol see Methods).

	Distance (Å)				
	I	*mode 1* (SM1)	*mode 1* (SM1)'	I	*mode 2* (SM2)
Cu1-Cu2	3.8	4.7	4.4	3.7	5.9
Cu1-H450	2.1	1.9	2.0	2.1	1.9
Cu1-H455	2.1	1.9	2.0	2.2	1.9
Cu1-E508	2.0	1.7	1.8	1.9	1.8
Cu2-E508	2.2	1.7	1.7	2.5	4.3
Cu2-H568	2.2	1.9	1.9	2.1	2.0
Cu2-E316				2.6	1.8/2.6
Cu2-Y318	2.2	1.8	1.9		
Cu2-OH^-					
Cu1-OH^-	2.0'		1.7		
Cu2-E329					
Cu1-w1				2.0	1.9
Cu2-w1				2.4	1.9
Cu2-w1				2.6	1.9

Table S10. DPP III MD simulations of DPP III structures with 2 copper ions bound into the substrate binding site and solvated in water Na^+ solution (Na^+ ions are added for the purpose of system neutralization).

Cu binding mode	Simulation label (structure name + replica number)	Time (ns)
Mode 1	SM1-1	300
	SM1-2	500
	SM1-3	300
Mode 1-OH⁻	SM1’-1	300
	SM1’-2	200
Mode 2	SM2-1	1200
	SM2-2	300
	SM2-3	1000

Table S11. Average LIE energies (electrostatic + vdw) kcal/mol during the indicated simulation time

	Average P-2Cu	Average PCu1-Cu2	Average P - Cu1
SM1-1 0.3 µs	-121 ± 6	-45 ± 3	-60 ± 4
SM1-2 0.5 µs	-123 ± 8	-46 ± 5	-61 ± 8
SM1-3 0.3 µs	-94 ± 14	-42 ± 4	-40 ± 12
SM1'-1 0.3 µs	-95 ± 8	-20 ± 6	-53 ± 3
SM1'-2 0.2 µs	-112 ± 7	-31 ± 5	-55 ± 3
SM2-1 1.2 µs	-113 ± 5	-58 ± 6	-55 ± 3
SM2-2 0.3 µs	-115 ± 15	-53 ± 15	-61 ± 3
SM2-3 1.0 µs	-107 ± 8	-52 ± 13	-55 ± 3

Suplementary Figures

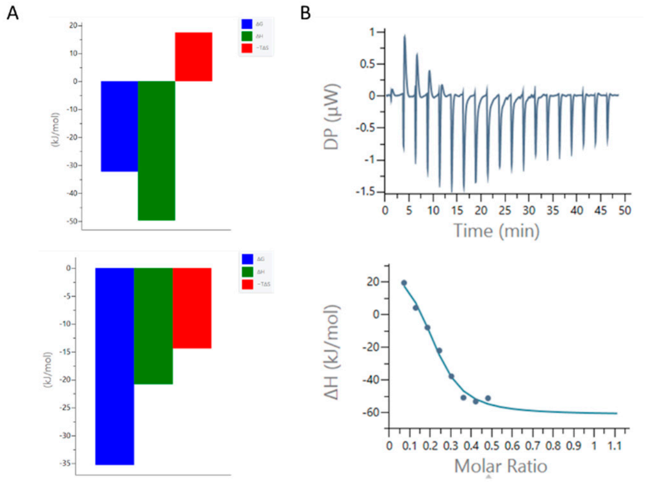

Figure S1. Inconsistent ITC for Cu^{2+}. A) Signature plots for direct (upper panel) and reverse (lower panel) titration of copper (II) metal ions and hDPP III; B) Additional binding site identified in reverse titration, with endothermic binding.

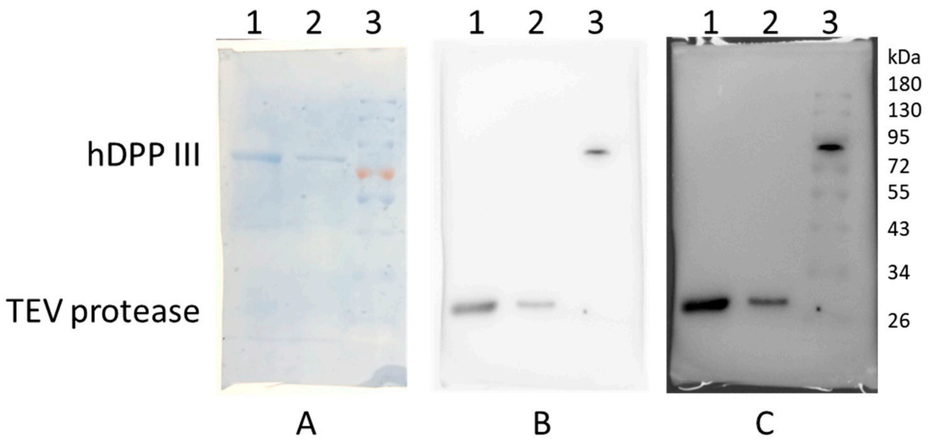

Figure S2. A representative anti His-tag western blot result. The membrane contains a sample of purified DPP III used for ITC measurements, with 2000 ng in lane 1 and 1000 ng in lane 2, and PageRuler protein marker in lane 3 with 100 ng of His-tagged hDPP III as reference. Plots A-C show the same membrane, first coloured by amido black staining (A), the result of the chemiluminescence under 1 min 15 s exposition (B), and the overlay figure showing the position of detected bands with the prestained markers (C).

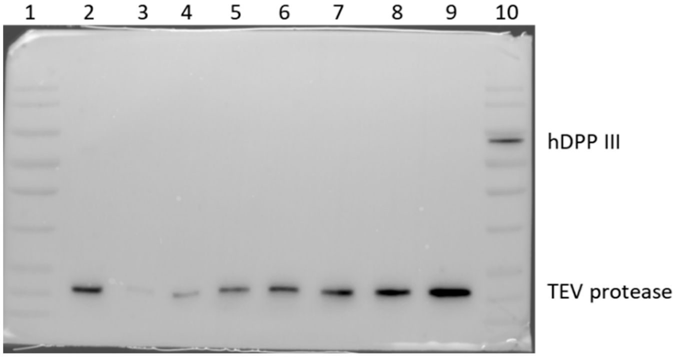

Figure S3. A representative western blot for TEV protease quantification. Chemiluminescent detection after 1 min 15 s exposition, overlaid with marker. Lanes: 1 - PageRuler prestained marker, 2 - 1000 ng of hDPP III ITC sample, 3 - 10 ng of TEV protease, 4 - 25 ng of TEV protease, 5 - 50 ng of TEV protease, 6 - 1000 ng of hDPP III ITC sample, repeated, 7 - 75 ng of TEV protease, 8 - 100 ng of TEV protease, 9 - 200 ng of TEV protease, 10 - PageRuler prestained marker with 100 ng of His-tagged hDPP III as reference.

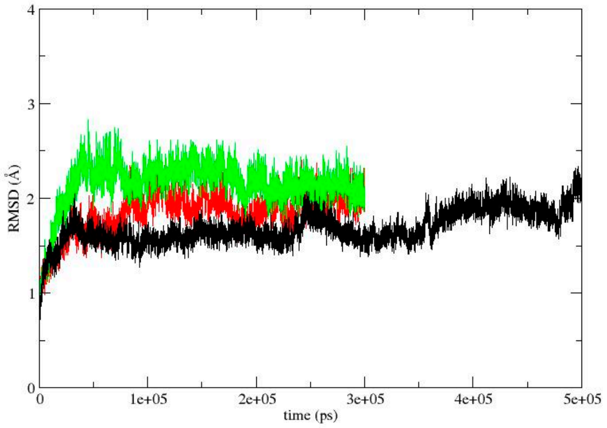

a)

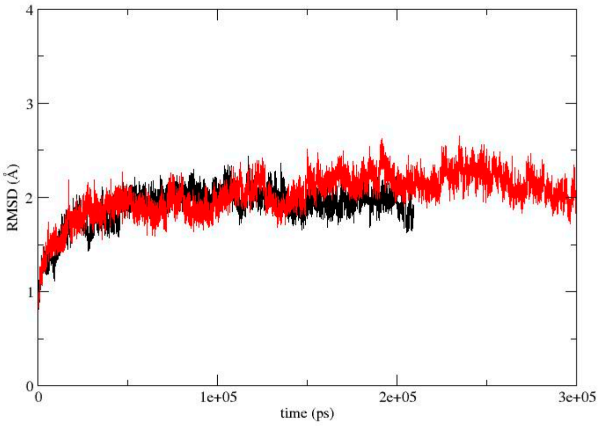

b)

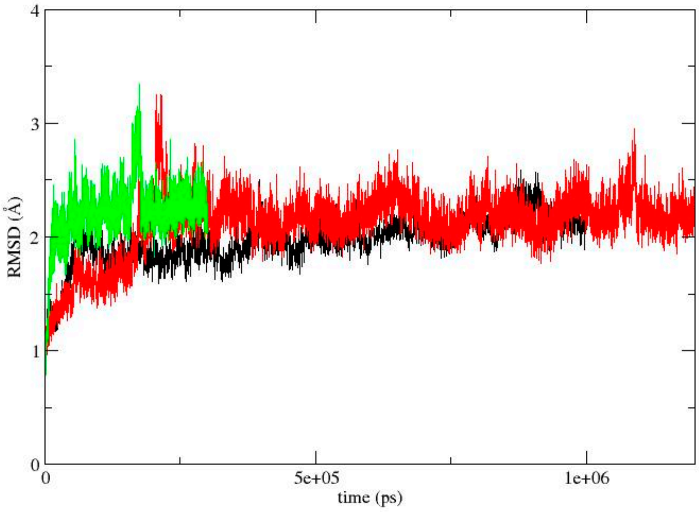

c)

Figure S4. RMSD of the protein backbone during MD simulations of DPP III structure with cooper ions bound in *mode 1* a) SM1-1 (red), SM1-2 (black), and SM1-3 (green), *mode 1'*, b) SM1'-1 (red), SM1'-2 (black), and in *mode 2*, c) SM2-1 (red), SM2-2 (green), and SM2-3 (black).

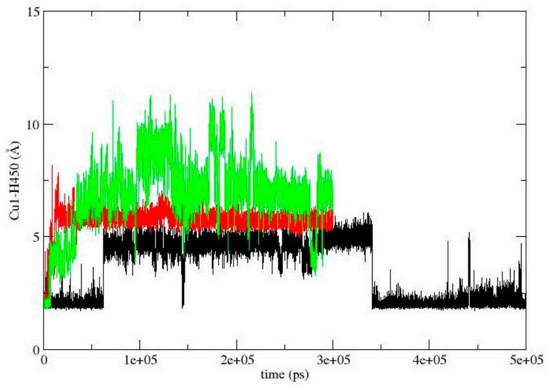

a)

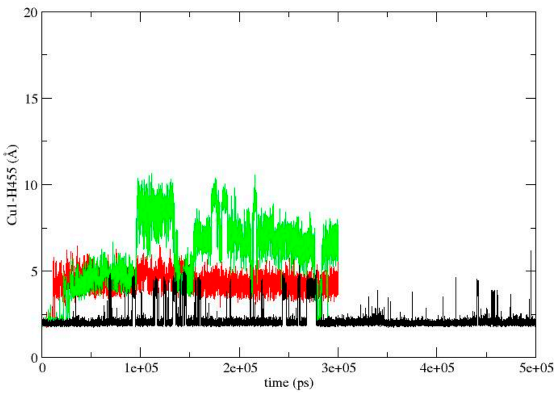

b)

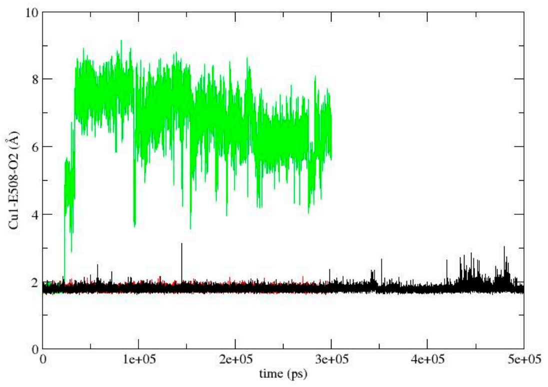

c)

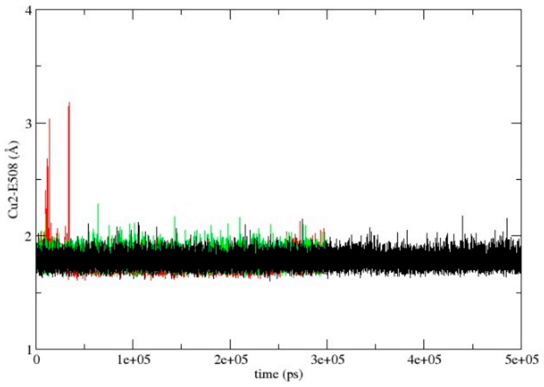

d)

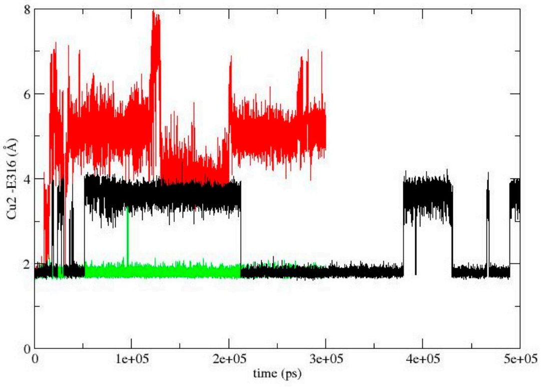

e)

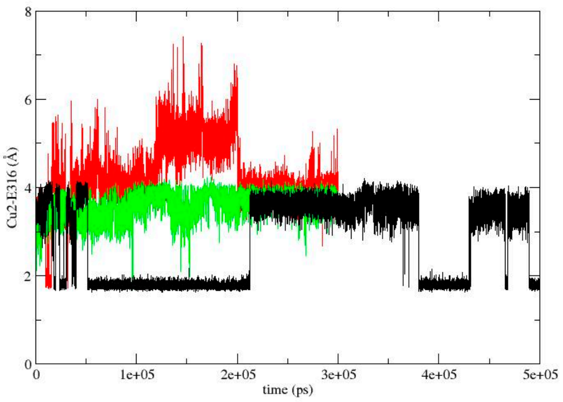

f)

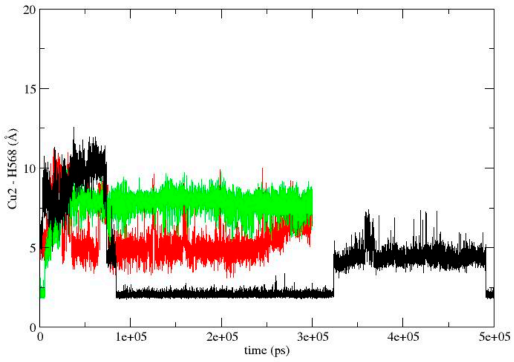

g)

Figure S5. Coordination of Cu1 (a-c) and Cu2 (d-g) during MD simulations of SM1 replicas, SM1-1 (red), SM1-2 (black), and SM1-3 (green)

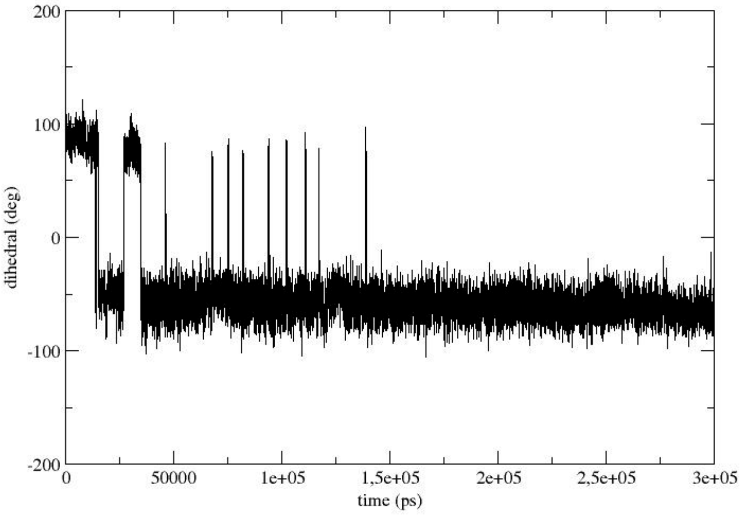

Figure S6. Values of the dihedral Cα-Cβ-Cγ-Cδ dihedral of E508 during MD simulations of SM1 replica SM1-1.

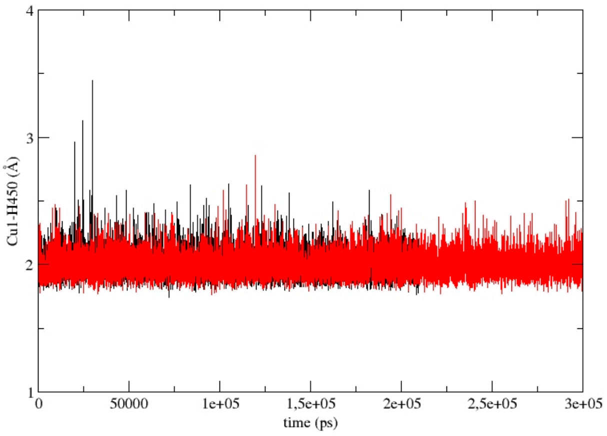

a)

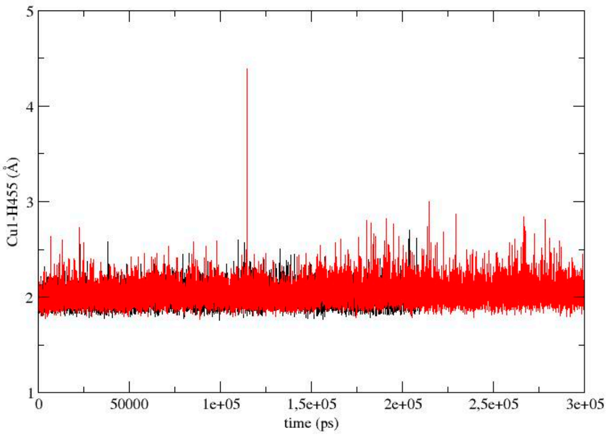

b)

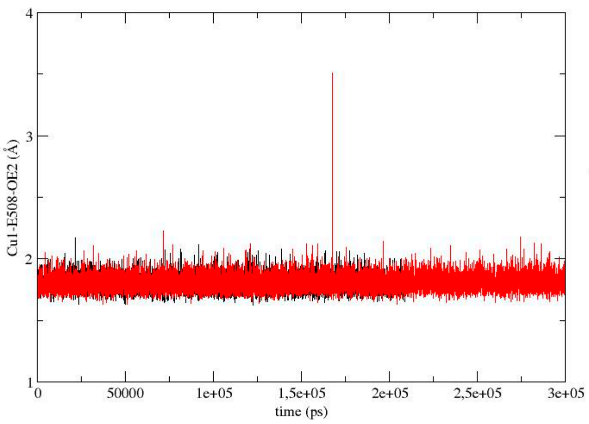

c)

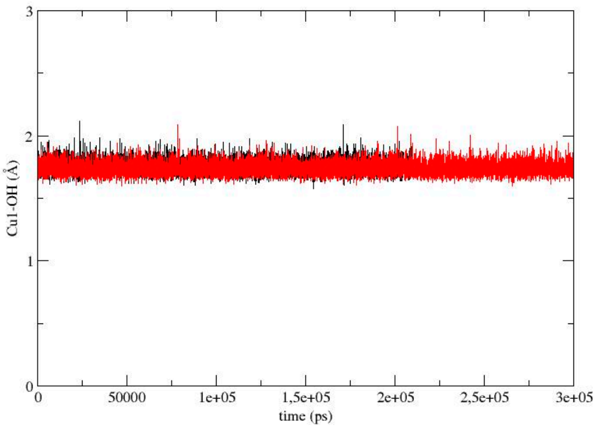

d)

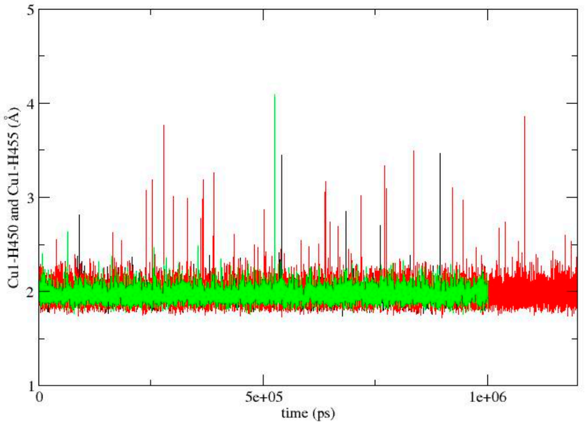

e)

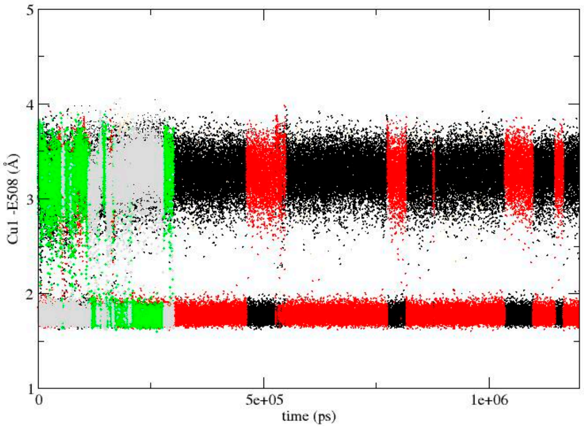

f)

Figure S7. Coordination of Cu1 during MD simulations of SM1’ replicas (a-d; SM1’-1, coloured black, and SM1‘-2, coloured red) and SM2 replicas (SM2-1, SM2-2, and SM2-3) (e-f; SM2-1 red, SM2-2 green, SM2-3 black).

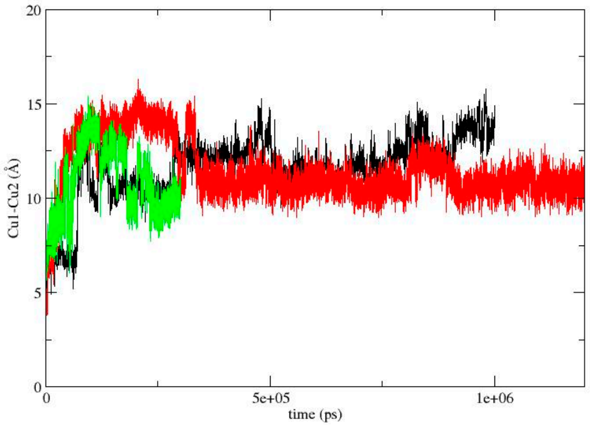

Figure S8. distance between Cu1 and Cu2 in simulations of SM2 replicas. Data related for different simulations of SM2 replica are represented by colours SM2-1 red, SM2-2 green, SM2-3 black.

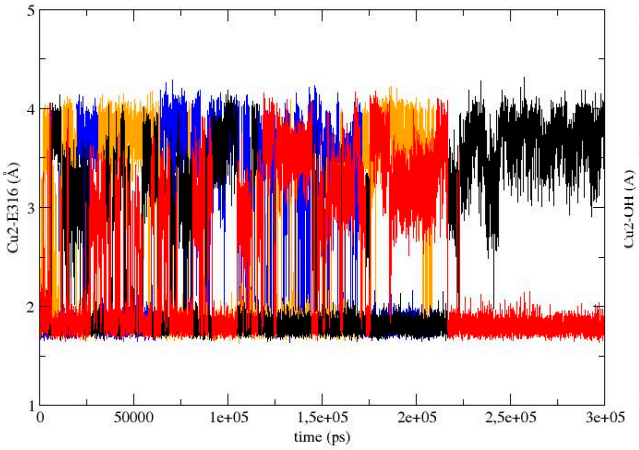

a)

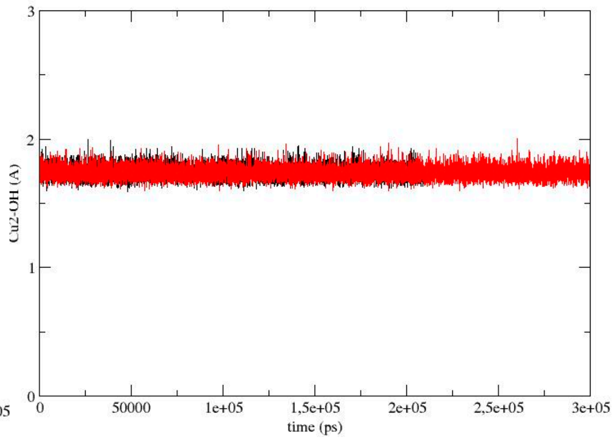

b)

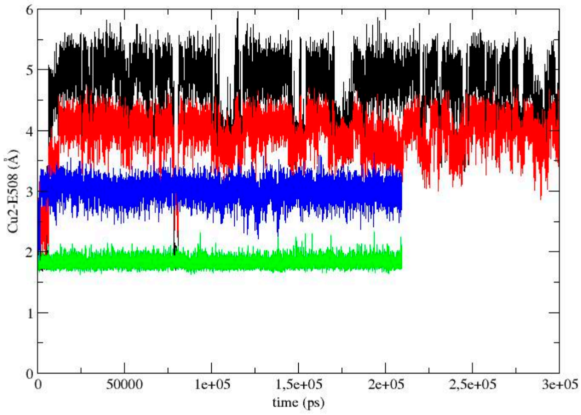

c)

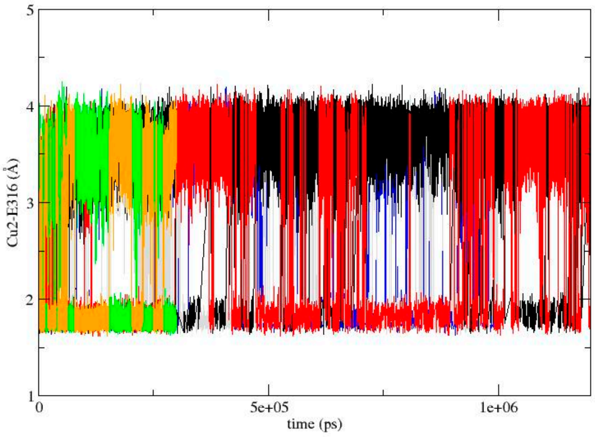

d)

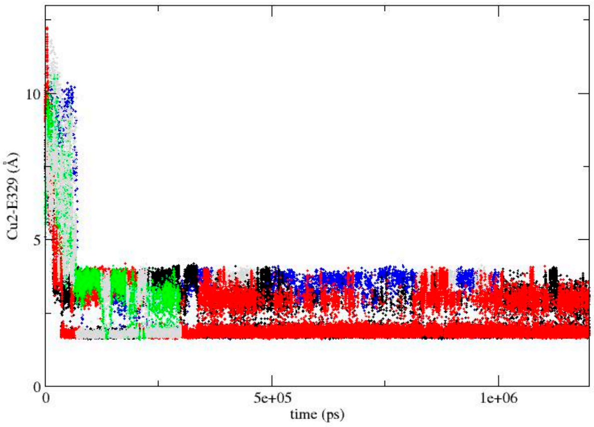

e)

Figure S9. Coordination of Cu2 during MD simulations of SM1' replicas (a-c; SM1'-1, coloured black, and SM1'-2, coloured red) and SM2 replicas (SM2-1, SM2-2, and SM2-3) (d-e; SM2-1 red & black, SM2-2 green & blue, SM2-3 yellow & grey)

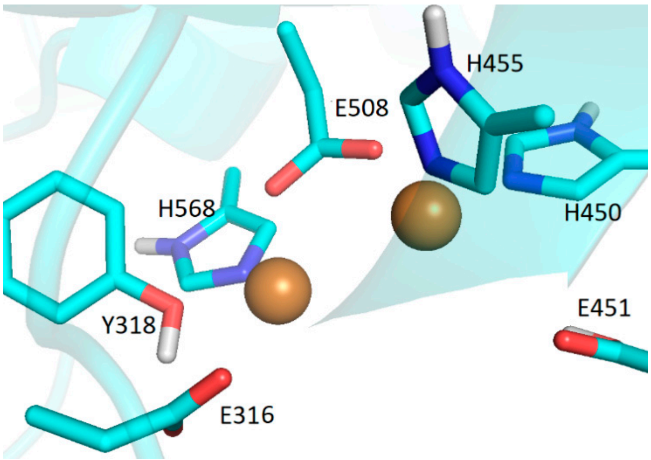

a)

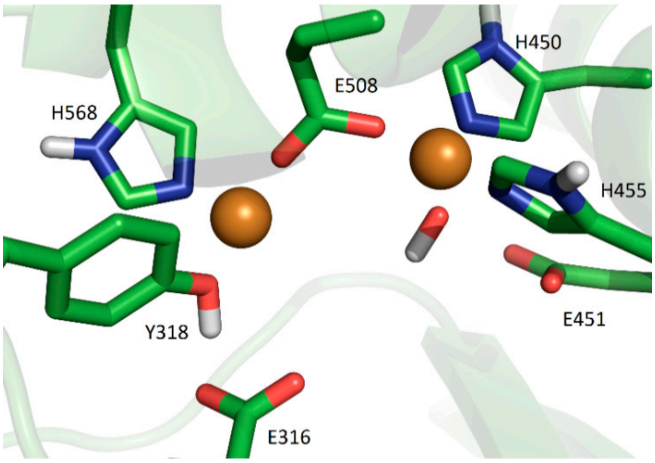

b)

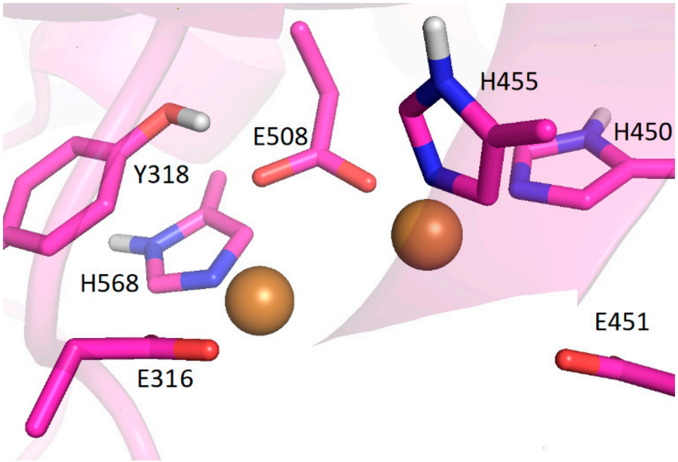

c)

Figure S10. Initial structures of DPP III after optimization used in MD simulations. The binding modes of Cu ions in DPP III are shown, a) DPP III-*mode 1* (SM1), b) DPP III-*mode 1*' (SM1'), c) DPP III-mode 2 (SM2). Cu ions are shown as spheres, and O from OH^- and amino acid residues coordinating metal ions as stick.
